# PAF Complex Plays Novel Subunit-Specific Roles in Alternative Cleavage and Polyadenylation

**DOI:** 10.1371/journal.pgen.1005794

**Published:** 2016-01-14

**Authors:** Yan Yang, Wencheng Li, Mainul Hoque, Liming Hou, Steven Shen, Bin Tian, Brian D. Dynlacht

**Affiliations:** 1 Department of Pathology, New York University (NYU) School of Medicine, New York, New York, United States of America; 2 Department of Microbiology, Biochemistry and Molecular Genetics, Rutgers New Jersey Medical School, Newark, New Jersey, United States of America; 3 Department of Biochemistry and Molecular Pharmacology, Genome Technology Center, New York University (NYU) School of Medicine, New York, New York, United States of America; Massachusetts General Hospital, Howard Hughes Medical Institute, UNITED STATES

## Abstract

The PAF complex (Paf1C) has been shown to regulate chromatin modifications, gene transcription, and RNA polymerase II (PolII) elongation. Here, we provide the first genome-wide profiles for the distribution of the entire complex in mammalian cells using chromatin immunoprecipitation and high throughput sequencing. We show that Paf1C is recruited not only to promoters and gene bodies, but also to regions downstream of cleavage/polyadenylation (pA) sites at 3’ ends, a profile that sharply contrasted with the yeast complex. Remarkably, we identified novel, subunit-specific links between Paf1C and regulation of alternative cleavage and polyadenylation (APA) and upstream antisense transcription using RNAi coupled with deep sequencing of the 3’ ends of transcripts. Moreover, we found that depletion of Paf1C subunits resulted in the accumulation of PolII over gene bodies, which coincided with APA. Depletion of specific Paf1C subunits led to global loss of histone H2B ubiquitylation, although there was little impact of Paf1C depletion on other histone modifications, including tri-methylation of histone H3 on lysines 4 and 36 (H3K4me3 and H3K36me3), previously associated with this complex. Our results provide surprising differences with yeast, while unifying observations that link Paf1C with PolII elongation and RNA processing, and indicate that Paf1C subunits could play roles in controlling transcript length through suppression of PolII accumulation at transcription start site (TSS)-proximal pA sites and regulating pA site choice in 3’UTRs.

## Author Summary

Gene transcription can be regulated through multiple mechanisms, such as histone modifications that create structural changes of the chromatin leading to gene activation or suppression, or regulation of the 3’ cleavage site of the mRNA, known as alternative cleavage and polyadenylation (APA), resulting in the generation of transcript isoforms with various lengths. Here we present genome-wide subunit-specific roles of the PAF complex (Paf1C) related to both mechanisms of transcriptional regulation. Using mouse muscle cells, we show contrasting results with yeast, namely, that depletion of Paf1C subunits does not affect certain histone modifications previously associated with this complex and that the complex exhibits subunit-specific functions. We also discovered a novel role of Paf1C in APA, wherein genome-wide transcript shortening occurs after depletion of three of the subunits. However, APA varies after depletion of certain subunits, reinforcing our conclusions regarding subunit specificity. Furthermore, by comparing depletions of two subunits, we show that the accumulation of RNA polymerase II (PolII) near the transcription start site (TSS) is specifically associated with the activation of TSS-proximal pA sites observed in one depletion but not the other.

## Introduction

### Diverse roles for Paf1C

Paf1C has been intensively explored in yeast, flies, and mammalian cells, which has led to diverse, and sometimes differing, conclusions. The complex was first characterized in yeast as a PolII-associated factor, and extensive use of mutants revealed that it plays a role in transcriptional elongation and chromatin modifications [1]. Mammalian Paf1C consists of six subunits (Paf1, Cdc73, Leo1, Ctr9, Rtf1, and Ski8) [2, 3]. Early studies suggested that Paf1C is an elongation factor, and indeed, Paf1C was shown to facilitate elongation [4, 5]. In contrast, very recent studies in flies and mammalian cells suggest that Paf1 could play a role in PolII pausing [6]. Paf1C is recruited through multiple contacts with the transcription machinery. For example, human Paf1, and to a lesser extent Leo1, bind PolII, whereas Ski8 is more peripheral, and Rtf1 weakly associates with Paf1C [3, 7]. In addition to its interactions with PolII, Ctr9 and Rtf1 were shown to be recruited through Spt6 and Spt5, respectively [8–10]. In human cells, promoter-bound trans-activators can also recruit Paf1C [11].

Robust data suggest that Paf1C plays an important role in acquisition of transcription-associated histone modifications: specific subunits (including Paf1 and Rtf1) were shown to be required to promote H2B ubiquitination (H2Bub), as well as H3 methylation at K4, K36, and K79 in yeast, flies, and humans [2, 7, 12–16]. However, the contribution of each subunit to chromatin modifications on a genome-wide scale has not been investigated. Furthermore, specific subunits are likely to play more or less extensive roles in generating these marks [4, 16], and these contributions may be context-dependent.

Other roles have also been ascribed to Paf1C. For example, biochemical studies have indicated that Ski8, a component of the SKI complex known to interact with the exosome, also associates with Paf1C [2], suggesting that the activities of these complexes could be mechanistically linked. Furthermore, in yeast, mutations in *Rtf1*, *Paf1*, and *Ctr9*, but not *Leo1* or *Cdc73*, led to mRNA transcript lengthening and defects in 3’ end formation of snoRNA, which are not polyadenylated and whose ends are processed via the exosome in concert with other processing factors [17]. On the other hand, mutations in yeast *Cdc73* impacted 3’ end formation of mRNA [18]. Further, mutations in yeast Paf1 led to altered 3’ cleavage and polyadenylation (pA) site usage and increased abundance of two extended mRNAs [19]. Although these results suggested a role for yeast Paf1 in alternative cleavage and polyadenylation (APA) regulation of a limited set of genes, a genome-wide role for Paf1C and possible mechanistic connections with PolII elongation and transcript processing were not investigated. Nonetheless, these studies suggest that Paf1C forms a multi-functional platform with a diversity of functions, some of which may be linked to ensure the fidelity of histone modifications, 3’ end formation, and RNA processing. This complexity is likely to explain why Paf1C plays a critical role in maintenance of stem cell identity and differentiation [20–26].

### A possible role in alternative cleavage and polyadenylation

Well over half of all mammalian genes contain multiple pA sites that lead to transcript variants with distinct 3’UTRs or coding regions [27]. Alternative cleavage and polyadenylation (APA) is thus emerging as an important gene regulatory mechanism that is highly regulated under physiological and pathological conditions [28–30]. For example, mouse genes tend to express longer 3’UTRs as embryonic development proceeds, and during myogenic differentiation, use of more distal pA sites is up-regulated relative to proximal sites as cells differentiate from myoblasts to myotubes [31]. Since the 3’UTR plays important roles in mRNA stability, translation, and subcellular localization, APA within this region (3’UTR-APA) can have a major impact on mRNA metabolism. As an example, 3’UTR-APA regulates miRNA-mediated gene control in the context of cell proliferation [32, 33]. APA can also generate different coding sequences, thereby expanding the repertoire of proteins within a cell. In the mouse genome, for example, ~40% of genes exhibit APA in upstream introns and exons, leading to variants with different coding sequences [34]. APA is regulated in part by the cleavage/polyadenylation (C/P) machinery, which encompasses over twenty polypeptides in mammalian cells, including poly(A) polymerase (PAP), poly(A) binding protein (PABPN1), Symplekin, and four protein complexes: the cleavage and polyadenylation specificity factor (CPSF), cleavage stimulation factor (CstF), cleavage factors I and II (CFI and CFII) ([35]{Shi, 2015 #2354, [36]). In addition, various RNA-binding proteins and splicing factors have been implicated in APA regulation [37].

Studies have indicated possible genetic and biochemical interactions between Paf1C and factors required for C/P in yeast and mammalian cells. For example, it was shown that yeast Ctr9 could associate with the CPSF160 homolog [18]. Moreover, antibodies against Cdc73 immunoprecipitated multiple components of CPSF, CstF, and Symplekin from HEK293T cell extracts. Further, depletion of Cdc73 and Ctr9 either abolished C/P in an *in vitro* assay or led to aberrant transcript lengthening of a human gene [11, 38]. However, these observations were primarily made in yeast or *in vitro*, or they were based solely on investigations of a small number of individual genes. Thus, a general role for Paf1C in C/P has not been established.

Here, we have investigated Paf1C function on a genome-wide scale in mouse muscle cells. We find that Paf1C subunits exhibit distinct functions with respect to gene occupancy and chromatin modifications. Moreover, we find that Paf1C was enriched not only at promoters but also downstream of 3’ ends, in contrast with previous observations in yeast. Surprisingly, loss of certain Paf1C subunits, but not others, leads to changes in histone modifications, PolII accumulation over gene bodies, and altered pA site usage. Our results suggest a novel role for Paf1C in suppression of C/P, providing a mechanism underlying APA regulation in muscle cells.

## Results

### Paf1C subunits differentially partition to chromatin

**Fig 1:**
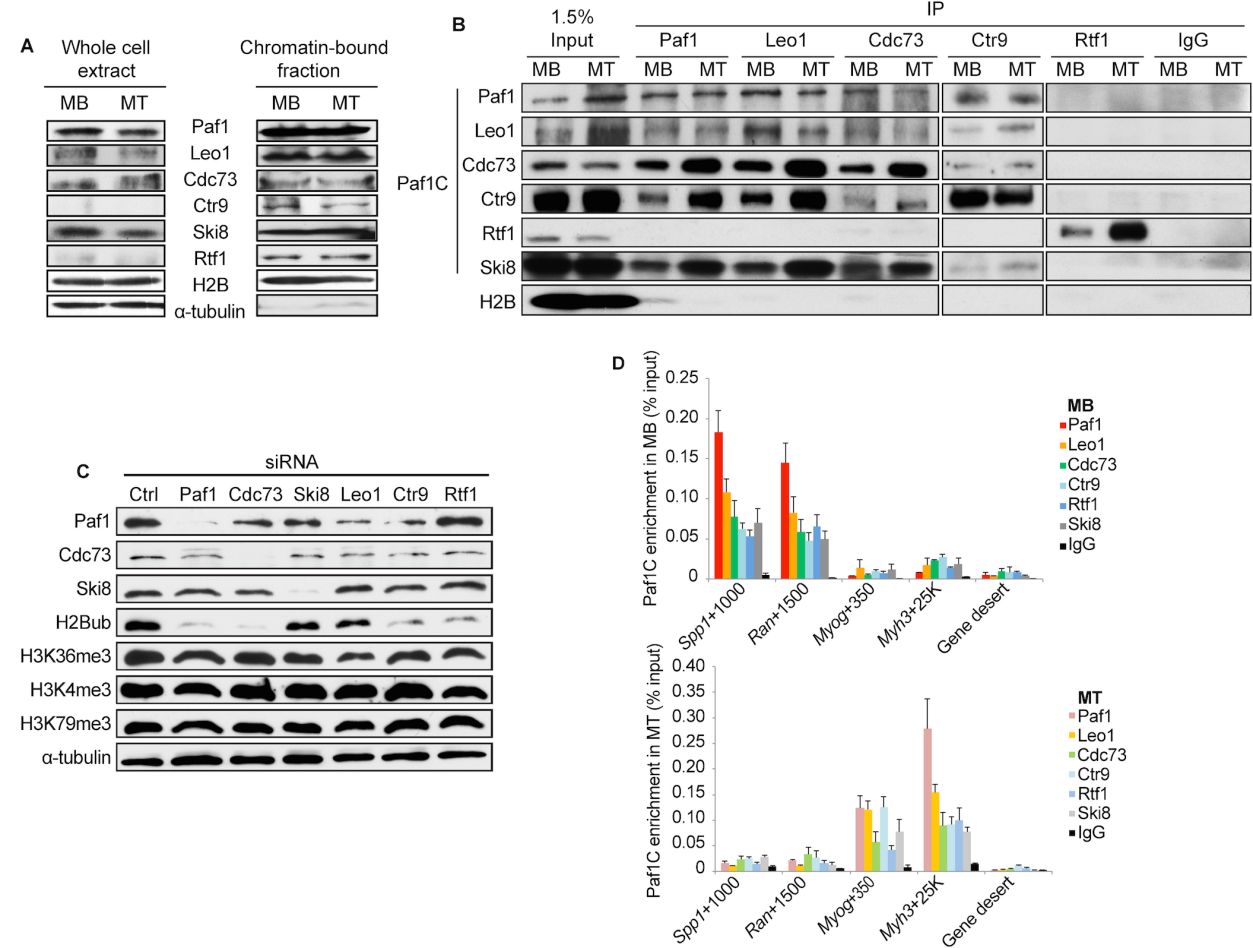
Paf1C subunits, except for Rtf1, are associated in mouse muscle cells but differentially impact histone modifications. **(A)** Western blot analysis of expression of Paf1C subunits in myoblasts (MB) and myotubes (MT). Rtf1 and Ctr9 are detectable in the chromatin-bound fraction but not whole cell extracts, as indicated. **(B)** Co-immunoprecipitation of endogenous Paf1C subunits, except for Rtf1, in the chromatin-bound fraction. IgG, control antibody. **(C)** siRNA-mediated depletion of the indicated Paf1C subunits in myoblasts have distinct effects on the levels of histone marks. siCtrl, non-specific control siRNA. **(D)** qChIP analysis of the enrichment of the Paf1C at specific loci in myoblasts and myotubes. The number shown for each locus indicates the midway point within the amplicon in base-pairs from the TSS of a specific gene. Gene desert is a genomic region devoid of genes.

We initiated our studies of Paf1C function in skeletal muscle, since the complex has not been extensively characterized in this tissue, and we made use of mouse C2C12 myoblasts, a well-established in vitro differentiation system. Our previous studies indicated that H2Bub dramatically decreased during myogenic differentiation, and this was due, in part, to reduced abundance of the enzyme (RNF20) that deposits this chromatin modification in differentiated myotubes [39]. Since Paf1C is known to recruit this enzyme to chromatin, we first examined expression levels for all subunits (Paf1, Leo1, Cdc73, Ctr9, Ski8, and Rtf1) in both whole-cell extracts and chromatin-bound fractions of C2C12 myoblasts (MB) and myotubes (MT). Essentially equivalent levels of expression were observed in both states for all subunits in the chromatin-bound fraction. Further, comparable levels of Paf1, Leo1, Cdc73, and Ski8 were observed in whole-cell extracts under both conditions, although Ctr9 and Rtf1 appeared to be depleted from these extracts as compared to chromatin (Fig 1A). These results suggest that specific Paf1C components can differentially partition to chromatin, whereas others may have additional nuclear roles apart from chromatin regulation.

To further investigate the interactions among Paf1C subunits, immunoprecipitation experiments were performed using solubilized chromatin from myoblasts or myotubes. Strikingly, we found that Rtf1 failed to co-precipitate with all other Paf1C subunits (Fig 1B). In parallel studies, we also ectopically expressed several components as amino- and carboxy-terminally tagged fusion proteins in myoblasts. Consistently, stable associations were observed between Cdc73 and Ski8, but not with Rtf1 (S1A Fig). The inability of Rtf1 to stably associate with the complex was also observed for human, *Drosophila*, and *S*. *pombe* counterparts [2, 3, 7, 14, 16, 40], although this result contrasts with budding yeast, wherein Rtf1 is an integral component of Paf1C [41].

Next, we investigated the impact of depleting Paf1C subunits on histone modifications thought to depend on various components of this complex. After verifying the depletion of each subunit, we detected H2Bub, H3K4me3, H3K36me3, and H3K79me3 (Figs 1C and S1B). Interestingly, we found that ablation of Paf1, Cdc73, Rtf1, and Ctr9 led to dramatic reductions in H2Bub, whereas no impact was observed after depletion of Ski8 or Leo1. In contrast, H3K4me3, H3K36me3 and H3K79me3 were not significantly affected by depletion of any subunit. These results extend our previous findings in skeletal muscle cells and in human tumor cells [7, 15, 39]. However, these findings diverge to some extent from observations in fission yeast, where depletion of multiple subunits led to significant decreases in H3K4me2/me3, and in other cancer cell lines, in which Leo1 silencing reduced H2Bub and Ski8 and Ctr9 depletion diminished H3K4me1/me3 and H3K79me2 [2, 4, 16]. These findings (1) reinforce our previous observations regarding uncoupling of H2Bub and H3K4me3 deposition in muscle cells [39] and (2) suggest that there may be species- and cell-type-specific differences in Paf1C function. Furthermore, the differences in fractionation and impact of ablation suggest that mouse Paf1C subunits are not tightly associated in a single complex, and they may have non-overlapping roles on chromatin, including distinct contributions to histone modifications. Thus, as in human cells [7], Rtf1 could localize to chromatin in a Paf1C-independent manner, potentially targeting different genes and performing distinct regulatory functions in skeletal muscle cells.

### Genome-wide localization of Paf1C reveals differences in subunit targeting during myogenesis

**Fig 2:**
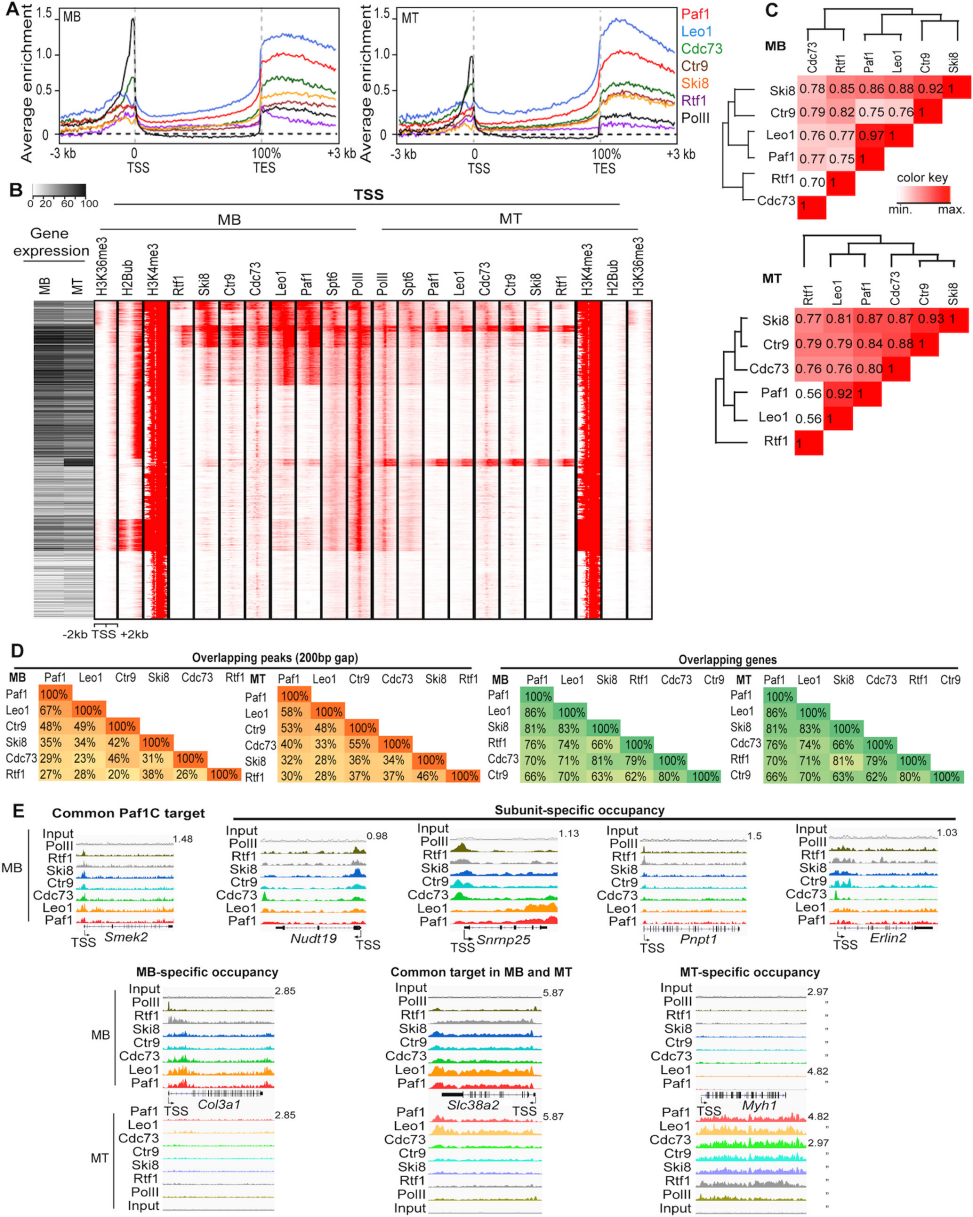
Genome-wide enrichment of Paf1C at both TSS and TES and subunit-specific localization on chromatin. **(A)** The average ChIP-seq enrichment profiles of indicated Paf1C subunits show occupancy across the gene with substantial enrichment at the transcription start site (TSS) and transcript end sites (TES). Enrichment (y-axis) is presented as the log_2_ ratio of RPM between ChIP and input samples in each 50 bp bin. **(B)** Left, A heatmap representing RNA-seq analysis of myoblasts and myotubes. Right, *k*-means clustering (*k* = 10) of ChIP-seq data reveals common and distinct localization of different Paf1C subunits. Spt6, histone modification and PolII data are also shown for comparison. Each row represents a single 4 kb genic region surrounding the TSS. The RNA-seq heatmap and ChIP-seq data are registered according to genes. **(C)** Cluster analysis of co-occupancy of Paf1C subunits on chromatin regions. Values are Pearson correlation coefficients. Correlations of RPM values encompassing genomic regions (TSS/TES upstream/downstream 1kb) were analyzed. **(D)** Percentage of overlapping ChIP-seq peaks or target genes of Paf1C subunits. Peaks within 200 bp from one another were considered overlapping. **(E)** Representative genome browser tracks illustrating Paf1C ChIP-seq data. The y-axis represents normalized reads in reads per million mapped (RPM). MB, myoblasts; MT, myotubes. Gene name and the maximum value of y-axis are indicated.

Intrigued by the possibility that Paf1C subunits might display unique activities, we comprehensively investigated the genome-wide occupancy of all six subunits in myoblasts and myotubes. To this end, we verified the specificity of each of our anti-Paf1C antibodies and confirmed their ability to enrich specific loci in chromatin immunoprecipitation (ChIP) experiments (Fig 1D). Next, we performed high-throughput sequencing after ChIP (ChIP-seq) and analyzed these data using a peak-finding algorithm (MACS). This global analysis resulted in several notable observations. First, we observed striking differences between the distribution of mouse Paf1C and yeast Paf1 [42]. Specifically, we found that within genic regions spanning 3 kb upstream of the TSS to 3 kb downstream of the transcript end sites (TES), the most pronounced enrichment of mouse Paf1C was found around the TSS and TES (Figs 2A and S2A). In contrast, yeast Paf1 exhibited the most significant enrichment over gene bodies, tapering off toward the TSS and TES, thus yielding a profile that was inverted with respect to that of the mouse complex [42]. Indeed, Paf1 showed markedly reduced enrichment within regions 100 bp downstream of the TES on yeast genes, whereas mammalian Paf1C exhibited strong binding to regions as much as several kb downstream of the TES (Fig 2A and 2E). This observation hints at potential functional differences between yeast and mouse Paf1C.

Moreover, average ChIP-seq enrichment profiles revealed notable differences among the subunits. For example, Cdc73 recruitment was most prominent near the TSS, and its profile closely resembled that of RNA PolII. On the other hand, Paf1 and Leo1 showed the strongest enrichment toward the 3’ ends of genes and downstream of the TES. Next, we used clustering to compare localization of the Paf1C subunits, PolII, and the elongation factor, Spt6, with histone modifications at regions surrounding the TSS and TES during myogenic differentiation (Figs 2B and S2B). We observed extensive co-localization of Paf1C and Spt6. Moreover, we observed preferential localization of specific subunits on TSS- or TES-proximal regions of individual genes. For example, certain clusters showed robust recruitment of all subunits except Rtf1 and/or Ctr9. Although we cannot rule out the formal possibility of differential accessibility of a given antibody for a Paf1C subunit, our data suggest that specific Paf1C components differentially localize to distinct sets of genes and sub-regions within genes.

We noticed that Paf1C occupancy was globally reduced as cells differentiated into myotubes, paralleling or exceeding the reductions observed for PolII recruitment (Figs 2B and S2B). Next, we compared Paf1C occupancy with gene expression in both myoblasts and myotubes. We found a general correlation between Paf1C occupancy and gene expression: genes expressed at moderate to high levels in myoblasts were nearly always bound by at least one component of Paf1C and exhibited robust levels of PolII, H3K4me3, H2Bub and H3K36me3, whereas genes that were not expressed or expressed at low levels exhibited negligible recruitment of any subunit and little enrichment of H2Bub or H3K36me3 (Figs 2B and S2B). Interestingly, we identified a group of genes with strong PolII binding over the TSS that exhibited insignificant levels of Paf1C occupancy, suggesting that on some genes, PolII alone may be insufficient to recruit this complex. We found that Paf1C recruitment generally decreased on many genes in myotubes, and many genes retained substantial levels of Cdc73 in the vicinity of the TSS, while lacking appreciable recruitment of the other Paf1C subunits. Despite the persistence of high levels of H3K4me3 and moderate to high levels of expression of many genes in myotubes, H2Bub was not detectable, as expected from our previous observation that the latter histone modification is not obligatorily linked to H3K4me3 during differentiation [39]. Our clustering analysis therefore strongly suggests that the loss of H2Bub in myotubes correlates with overall reduction of Paf1C, but no such correlation holds for H3K4me3 and H3K36me3, consistent with our biochemical analyses (Fig 1C). We note, however, that Paf1C binding is not strictly linked to H2B ubiquitylation, since many genes exhibit Paf1C binding without H2Bub, especially in myotubes.

### Paf1C target genes are involved in diverse biological processes

Next, we investigated the extent to which Paf1C subunits bound to common and unique sets of genes by systematically analyzing our ChIP-seq data (Fig 2C and 2D). Consistently, Paf1 and Leo1 occupancy showed the strongest concordance in both myoblasts and myotubes, whereas Cdc73, Ctr9, Rtf1, and Ski8 showed reduced levels of co-localization amongst themselves and with Paf1/Leo1 targets. Strikingly, the percentage of genes common to multiple Paf1C subunits far surpassed the percentage of overlapping peaks, reflecting differences in binding sites within the same gene (Fig 2D and 2E). Several lines of evidence suggest that these differences are meaningful and could not be ascribed to sequencing depth or inter-experimental variation. First, in each case, we sequenced >50 million tags per factor, a number that generally yields sufficient coverage in ChIP-seq analysis of transcription factors in our experience, then normalized all data with respect to the total number of sequence tags and compared their reads per million (RPM) across all ChIP-seq experiments. Second, when we analyzed biological replicates for each Paf1C subunit and compared them with ChIP-seq data from all other subunits (analogous to Fig 2C), we found consistently stronger correlations between ChIP-seq replicates than between different Paf1C subunits (S2D Fig). Third, we identified clusters that showed enrichment for multiple factors on TSS-proximal regions that were devoid of other factors altogether in both conditions (Fig 2B). Indeed, we verified a subset of subunit-specific peaks identified by ChIP-seq using ChIP coupled with quantitative PCR (ChIP-qPCR) (Figs 2E and S2B). By comparing subunit co-occupancy in myoblasts and myotubes, we also noted myogenesis-associated changes (Fig 2C, 2D and 2E). To investigate the biological functions of Paf1C target genes, we performed gene ontology (GO) analysis (S3A Fig). Notably, we found that target genes bound by all six Paf1C components in myoblasts were significantly enriched for ontologies associated with regulation of transcription and RNA processing. Interestingly, “RNA processing” was also the most over-represented GO category among genes bound only by Paf1 and Leo1, but not for genes bound exclusively by Ski8 or Rtf1. This finding indicated that Paf1 and Leo1 may be more closely linked to the regulation of this process (S3A Fig). In addition to the unique biological processes associated with targets of different Paf1C subunits, subunit-specific differences were also observed as a function of differentiation (S3A Fig). For example, cell cycle genes were the most over-represented class of Paf1 target genes in proliferating myoblasts, whereas in myotubes, Paf1 relocated from these genes and was instead recruited to muscle development genes. This reorganization correlated with changes in gene expression profiles during myogenesis and the tendency for Paf1C to localize to highly transcribed genes (Figs 2B and S2B). Nonetheless, RNA processing genes were commonly bound by Paf1 in both myoblasts and myotubes (S3A Fig). This list encompassed a large number of genes encoding splicing factors, as well as other rRNA and mRNA processing factors. In addition, many components of the Integrator complex, which is involved in PolII elongation and 3’ end processing of small RNAs, were bound by Paf1C. Taken together, these data reinforce the conclusion that Paf1 and Leo1 are the most tightly associated components—both physically and functionally—whereas the other four subunits showed less coherent patterns of recruitment to target genes. Thus, on a genomic level, Paf1C subunits are likely to act distinctly to control diverse biological processes.

### Paf1C plays roles in gene expression and alternative cleavage and polyadenylation

To investigate potential functional differences among Paf1C subunits, we individually ablated Paf1, Cdc73, and Ski8, since our analyses of factor binding in myoblasts suggested that these proteins might belong to distinct sub-clusters (Fig 2C). Through transcriptome profiling using RNA-seq, we observed that genes were both up- and down-regulated by Paf1 depletion. Further, clustering analysis indicated that Paf1 and Cdc73 silencing resulted in de-regulation of a common set of targets, whereas Ski8 knock-down led to aberrant expression of a largely non-overlapping set of genes (S3B Fig). GO analysis showed that mitotic cell cycle genes were down-regulated after ablation of Paf1 or Cdc73, whereas certain RNA processing genes were up-regulated upon silencing Paf1, Cdc73, or Ski8 (S3C Fig). These results reinforce the notion that subunits within this complex play diverse roles in skeletal muscle.

**Fig 3:**
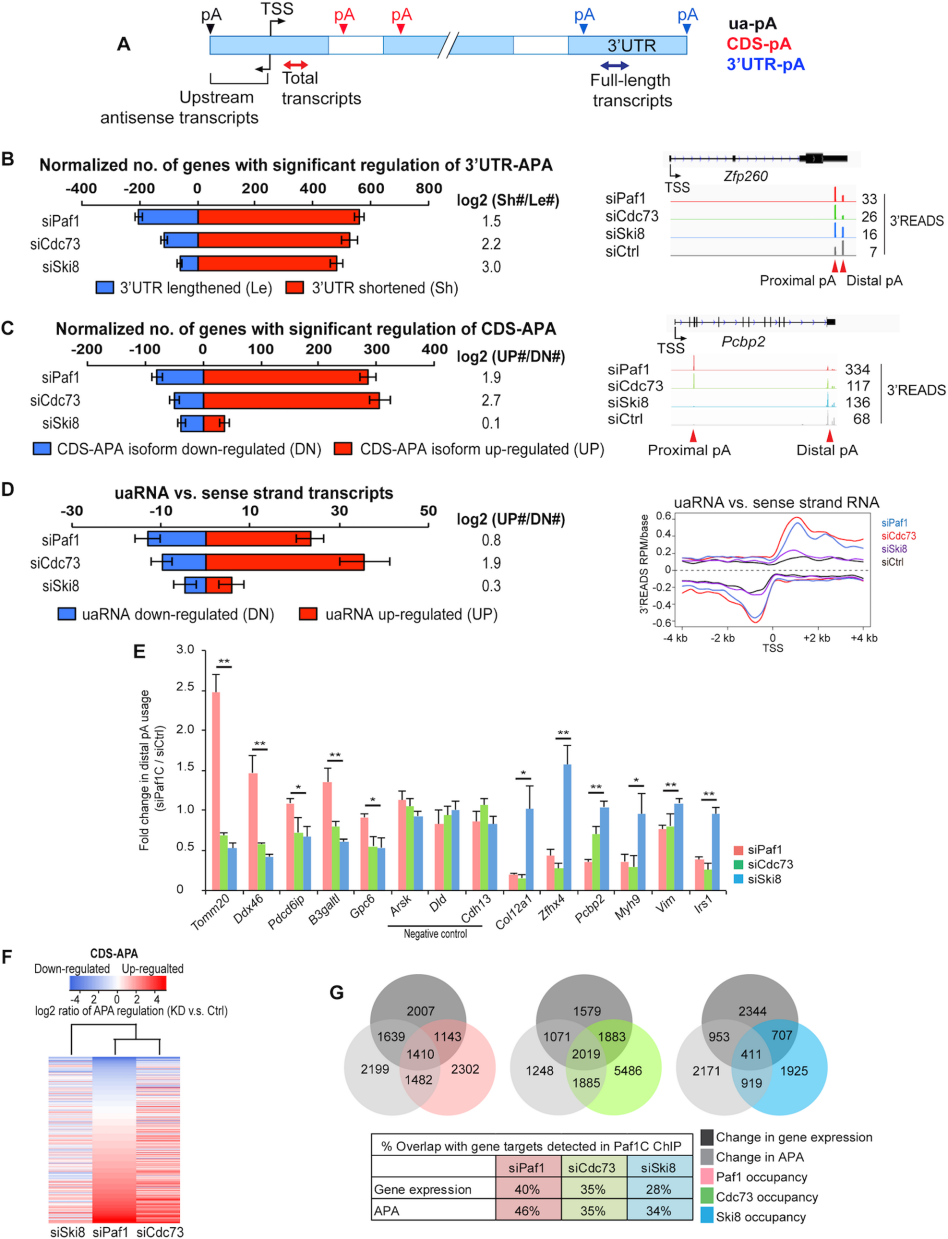
Depletion of Paf1C subunits leads to genome-wide changes in pA site usage. **(A)** Schematic depicting different types of APA sites and method to detect total and full-length transcript abundance. Solid boxes are exons, and open ones are introns. 3’UTR-APA sites are those located in the 3’UTR. CDS-APA sites are those located in introns or exons upstream of the 3’-most exon. **(B)** Left, normalized number of genes with regulated 3’UTR-APA in each sample as examined by Global Analysis of Alternative Polyadenylation (GAAP, see Materials and Methods for detail). Normalized number is based on (observed value—expected value). Red and blue bars represent genes with shortened 3’UTRs (Sh, proximal pA isoform up-regulated relative to distal pA) and lengthened 3’UTRs (Le, distal pA isoform up-regulated relative to proximal pA), respectively. FDR = 0.05 (Significance Analysis of Alternative Polyadenylation, SAAP, see Materials and Methods for detail) was used to select genes with significant 3’UTR-APA regulation. Only the top two most abundant APA isoforms (based on the number of PASS reads) of each gene were used for this analysis. Error bars show standard deviation based on 20 iterations of bootstrapping. Log2(Sh#/Le#) is log2(ratio) of the number of Sh genes to the number of Le genes. Right, genome browser tracks showing 3’READS data for a selected gene, *Zfp260*. The maximum value of y-axis for each track is indicated. APA sites are indicated by arrows. (**C**) Left, normalized number of genes with regulated CDS-APA as examined by GAAP. Red and blue bars represent genes with up-regulated CDS-APA isoforms (UP) and down-regulated isoforms (DN), respectively. All CDS-APA isoforms were combined and compared to all 3’-most exon isoforms combined by SAAP. FDR = 0.05 (SAAP) was used to select genes with significant CDS-APA regulation. Error bars are as described in panel B. Samples were sorted by the total number of genes with CDS-APA changes. Log2(UP#/DN#) is log2(ratio) of the number of UP genes to the number of DN genes. Right, genome browser tracks showing 3’READS data for a selected gene, *Pcbp2*. The maximum value of y-axis for each track is indicated. APA sites are indicated by arrows. (**D**) Left, normalized number of genes with regulated uaRNA expression. Red and blue bars represent genes with up-regulated (UP) and down-regulated (DN) uaRNA expression, respectively. Log2(UP#/DN#) is log2(ratio) of the number of UP genes to the number of DN genes. All uaRNAs were combined and compared to all sense strand transcripts whose pAs were beyond 2 kb from the TSS by SAAP. Q-value < 0.05 (SAAP) was used to select genes with a significant uaRNA expression difference. Error bars are as described in panels B and C. Right, metagene plots of uaRNA and sense strand RNA expression in siPaf1, siCdc73, siSki8 and siCtrl samples. Expression is represented by reads per million (RPM, pA site-supporting reads only) at pA positions. **(E)** RT-qPCR validation of impact on APA after depletion of Paf1, Cdc73, or Ski8. *p*-value indicates significant difference between Paf1- and Ski8-depleted cells. Negative control genes are indicated. **(F)** Heatmap showing common and distinct target genes with changes in CDS-APA after depletion of Paf1, Cdc73 or Ski8. Genes were sorted using the siPaf1 data. Samples were clustered using Pearson correlation. **(G)** Top, Venn diagrams depicting overlap of genes among Paf1C targets as detected by ChIP-seq, genes showing altered levels of expression in RNA-seq (fold-change >1.4, *p*-value<0.01 (Fisher's exact test)) and genes with changes in APA after depletion of Paf1C subunits (SAAP FDR = 0.05; see Materials and Methods). Right, data are shown in percentage of overlap.

Given that Paf1 binding is enriched in the vicinity of the TES (Fig 2A and S2A Fig) and that some Paf1C subunits have been implicated in the regulation of C/P in yeast, we set out to test the effect of knocking down Paf1C subunits on pA site usage in C2C12 cells. To investigate pA site usage on a genomic scale, we knocked down Paf1 from myoblasts and applied 3' region extraction and deep sequencing (3'READS) [34] to poly(A)+ RNAs. In parallel, we also knocked down Cdc73 and Ski8 because of their distinct binding profiles on genic regions (Fig 2C). Each pA site detected by 3’READS was classified according to its location within a gene, namely, within internal exons or introns that affect the coding-sequence (CDS) or within the 3’-most exon affecting the 3’ untranslated region (3’UTR) [43] (Fig 3A). To mitigate the effect of different sequencing depths on the comparison, we applied the Global Analysis of Alternative Polyadenylation (GAAP) method [43], in which we randomly sampled the same number of pA site-supporting (PASS) reads from each sample (1.5M) and repeated this process 20 times to assess data variability (Fig 3B, 3C and 3D; see Materials and Methods for details). For the analysis of 3’UTR-APA, we selected the two most abundant pAs within the 3’UTR per gene based on read numbers and examined their relative expression levels. For CDS-APA analysis, we compared pAs located in the 3’-most exon of each gene with all pAs located in upstream introns/exons of the same gene. Using a false discovery rate (FDR) cut-off of 0.05, we identified significantly regulated APA events.

Remarkably, ablation of all three genes led to pervasive transcript shortening, although the locations of regulated APA sites differed significantly (Fig 3B and 3C). Within the 3’UTR, all three siRNA treatments led to dramatic up-regulation of proximal pA sites (Figs 3B and S5A), leading to shortening of 3’UTR length. Depletion of Paf1 or Cdc73 also significantly activated CDS-APA sites, resulting in a shift of pA usage from the 3’-most exon to upstream introns/exons (Fig 3C). In striking contrast, Ski8 knock-down led to considerably less activation of CDS-APA sites (Fig 3C). This result indicates that Ski8 plays different roles in APA than Paf1 and Cdc73. We also found that upstream antisense transcripts (uaRNAs) were significantly up-regulated after depletion of Paf1 or Cdc73, but not after silencing of Ski8 (Fig 3D). Note that uaRNAs are generally expressed at much lower levels than sense transcripts, resulting in reduced detection of significantly regulated events, as compared to the occurrence of 3’UTR-APA and CDS-APA, when the same sequencing depth and FDR were used in this analysis (Fig 3B, 3C and 3D).

Using a quantitative reverse-transcription-coupled PCR (qRT-PCR) method, we validated our 3’READS results for several genes (Fig 3E). Importantly, with respect to CDS-APA profiles, when we compared the gene targets de-regulated upon loss of each Paf1C subunit, we found that knock-downs of Paf1 and Cdc73 shared the greater number of common targets as compared to knock-down of Ski8 (Fig 3F). Overall, we found that Paf1 occupancy strongly corresponded with regulation of expression or APA usage after knock-down, with 40 or 46% of target genes showing significant changes, respectively (Fig 3G). This correlation was slightly lower for Cdc73 (35%) and Ski8 (28 or 34%).

GO analysis indicated that genes that exhibited altered APA were enriched for several terms, including RNA splicing and gene silencing by RNA, confirming that Paf1C not only binds, but also regulates, targets involved in these biological processes (S4 Fig). Taken together, these findings suggested a novel role for Paf1C in pA site selection and prompted us to analyze in greater detail the impact of Paf1C subunits on APA.

### Silencing of Paf1C subunits up-regulates TSS-proximal intronic pAs

**Fig 4:**
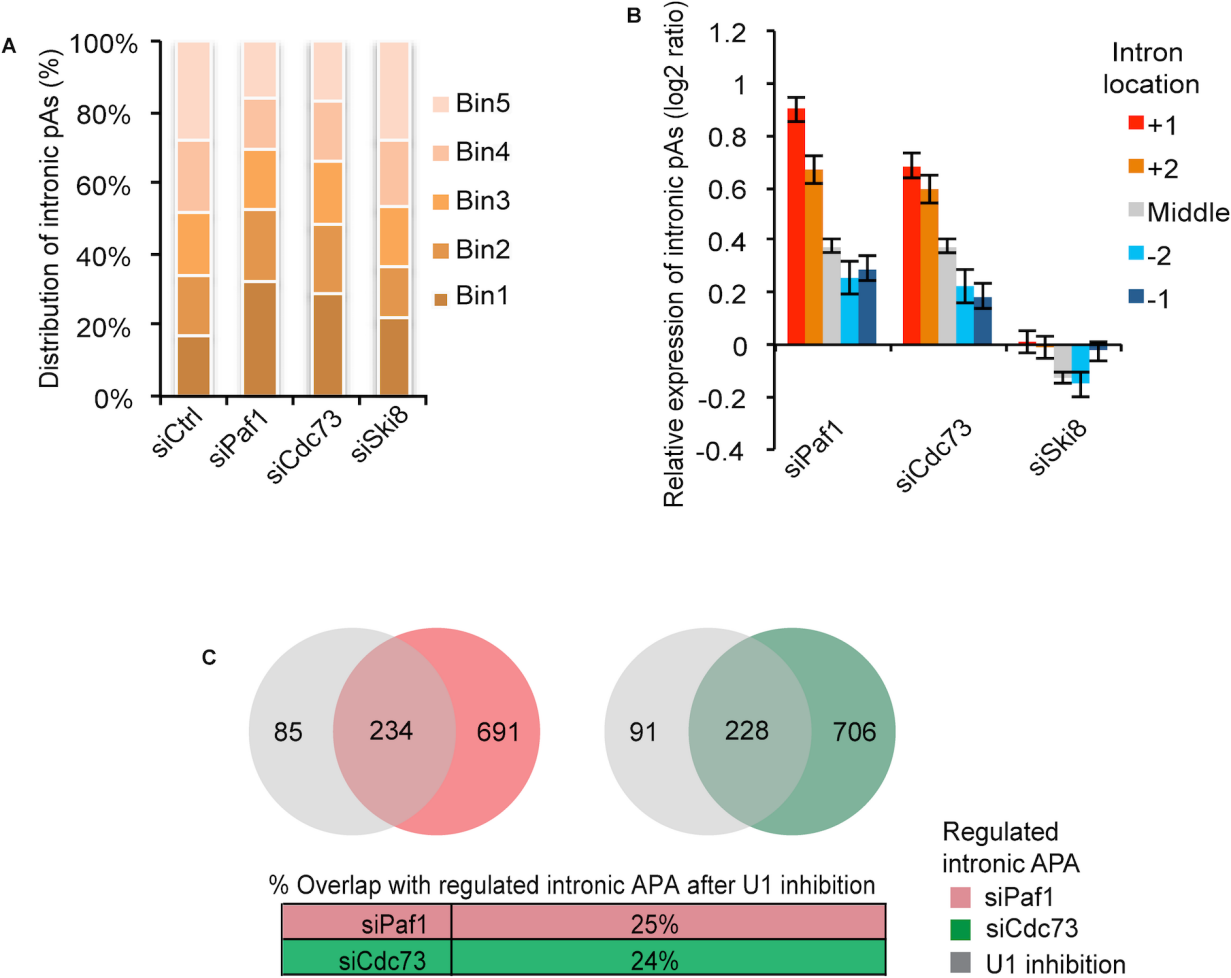
Depletion of Paf1, Cdc73 but not Ski8 leads to prevalent activation of TSS-proximal intronic APA sites. **(A)** Up-regulation of intronic APA sites is biased toward the TSS after depletion of Paf1 or Cdc73, but not Ski8. Regions between TSS and the 3'-most splice site were divided into 5 equally-sized bins. “siCtrl" indicates the background distribution of intronic pAs whose usage was detected in control cells. The distribution of up-regulated intronic pAs in knock-down samples were compared to background. The *p*-values (Fisher’s exact test) for the percentage of pAs in Bin1, the 5’-most bin, for each depletion and control pair are 4x10^-28^, 4x10^-19^, and 0.02 for siPaf1, siCdc73, and siSki8, respectively. **(B)** TSS-proximal intronic pAs are more strongly activated as compared to downstream intronic pAs. +1, +2, middle, -2 and -1 introns are the first, second, middle (not first two or last two), second to the last, and the last introns, respectively. The error bar represents standard error of mean for all analyzed pAs in a given intron group. Expression changes are log2(ratio) of PASS reads in knock-down sample vs. control sample. **(C)** Top, Venn diagrams depicting overlap of genes with regulated intronic pAs between Paf1- or Cdc73-depletion and U1 inhibition. Bottom, data are shown in percentage of overlap.

For regulated CDS-APA events, we focused on intronic pAs (accounting for >94% of the CDS-APA events; S5B Fig), and further examined their locations in genes. By dividing each genic region into five equally sized sub-regions, we found that up-regulated CDS-APA sites in both Paf1- and Cdc73-depleted cells were biased toward 5’-most regions (32.4% and 29.1% in Bin 1) as compared to the control (17.2% in Bin 1). Both biases were statistically significant (*p*-value = 4×10^−28^ and 4×10^−19^ for Paf1 and Cdc73, respectively; Fig 4A). By contrast, the bias was very modest for Ski8 depletion (21.7% in Bin 1 and *p*-value = 0.02). The expression changes for intronic pA isoforms were plotted against their locations within either the first two TSS-proximal introns (+1 and +2, in order), the last two distal introns (-2 and -1), or intervening introns (M, Fig 4B). In the Paf1 knock-down, the first TSS-proximal intron exhibited the most significant increase in pA site usage. The occurrence of APA site up-regulation dropped sharply within the middle introns and distal introns. Interestingly, a similar correlation was observed after Cdc73, but not Ski8, silencing (Fig 4B). As expected, the CDS-APA profiles of Paf1- and Cdc73-depleted cells were much more similar compared to that of Ski8 knock-down (Fig 4C). Thus, despite the fact that ablation of all three proteins led to widespread transcript shortening, depletion of Paf1 or Cdc73 significantly activated TSS-proximal intronic APA sites, whereas Ski8 knock-down provoked shortening primarily within the 3’UTR (Fig 3B and 3C). Our results thus suggest a novel, genome-wide role for Paf1C as a suppressor of APA and further reinforce our conclusion regarding subunit-specific roles in gene regulation.

Recent studies have established that U1 snRNP, beyond its role in splicing, can suppress APA by preventing usage of proximal, cryptic pA sites [44–46]. Global analysis of the effect of suppression of U1 function in C2C12 myoblasts indicated that pA usage was preferentially up-regulated within the first two introns [43], similar to our findings in Paf1- and Cdc73-ablated cells. Therefore, we compared the set of genes regulated by U1 inhibition with targets showing aberrant intronic APA after Paf1 or Cdc73 depletion. Interestingly, we found that the lists of genes were largely non-overlapping (Fig 4C), since the majority of Paf1 or Cdc73 targets (75 or 76%, respectively) were not regulated by U1 inhibition. Although further studies are required, these results suggest that Paf1C-dependent mechanisms governing the regulation of CDS-APA may be largely distinct from U1-mediated suppression of cryptic, proximal pA site usage, possibly because the latter involves the interaction between U1 snRNA and 5’ splice site sequences, but the former does not.

### Paf1C is highly enriched on genes that are shortened upon Paf1 depletion

**Fig 5:**
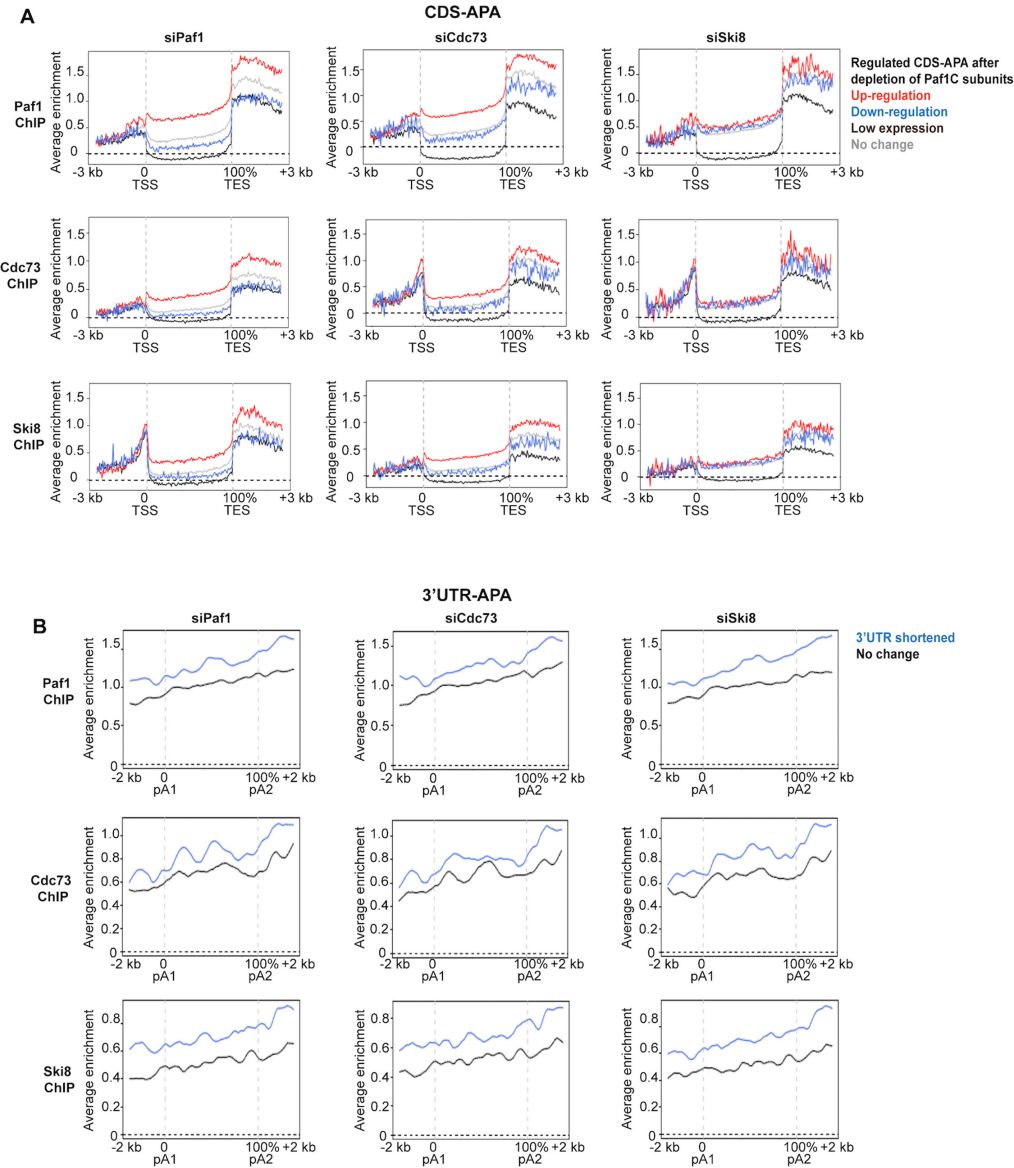
Chromatin occupancy of Paf1C correlates with APA regulation after depletion of Paf1C subunits. **(A)** The average enrichment profiles of Paf1, Cdc73, and Ski8 were plotted on genes that showed regulated, low-expressed, or unaffected CDS-APA after depletion of Paf1, Cdc73 or Ski8. A region from 3 kb upstream of TSS to 3 kb downstream of TES was analyzed. **(B)** The average enrichment profiles of Paf1, Cdc73, and Ski8 were plotted over genes that showed shortening or unaffected length of 3’UTR after depletion of Paf1, Cdc73, or Ski8. A region from 2 kb up-or downstream of the two most regulated 3’UTR pA sites was analyzed (A distance between the proximal and distal pAs greater than 1 kb was required).

To probe the mechanisms behind transcript shortening resulting from CDS-APA in Paf1- depleted cells, we first examined the enrichment of Paf1C subunits on the corresponding genes in wild-type myoblasts. Genes that showed significant up-regulation of CDS-APA sites were compared with all other classes, including clusters that are not affected or that exhibited down-regulation, as well as genes that are not expressed or expressed at low levels. We examined the average distribution profiles for all six Paf1C subunits on regions spanning from 3 kb upstream of the TSS to 3 kb downstream of the TES. In general, Paf1C subunits were depleted from the bodies of genes exhibiting low expression (Fig 5A). In contrast, we found that all six Paf1C subunits were enriched on genes producing prematurely shortened transcripts in the Paf1 knock-down (Figs 5A and S5C). A similar enrichment of Paf1C subunits was also observed on genes exhibiting changes in APA sites after silencing of Cdc73. Importantly, however, this effect was subunit-specific, since we did not observe enrichment for any Paf1C subunits on genes exhibiting changes in CDS-APA upon Ski8 silencing (Figs 5A and S5C). These results are consistent with the observation that Ski8 ablation resulted in substantially less regulation of TSS-proximal pA sites than Paf1 and Cdc73 depletion. We also investigated Paf1C occupancy around 3’UTR pA sites, since depletion of all three Paf1C subunits resulted in significant 3’UTR shortening (Fig 3B). In contrast with our observations on CDS-APA sites, we found that all Paf1C subunits, including Ski8, were enriched around 3’UTR pA sites on genes that exhibited 3’UTR shortening after depletion of Paf1, Cdc73, or Ski8 (Figs 5B and S5D). Altogether, these data suggest a potential protective mechanism, whereby occupancy by Paf1C serves to prevent 3’ end processing at proximal pA sites. Further, these results suggest diversification of function within the Paf1C complex: on one hand, Paf1 and Cdc73, but not Ski8, are critical for directly regulating usage of TSS-proximal pA sites, whereas APA at distal sites (3’UTR) may be regulated by a distinct complex that minimally includes Paf1, Cdc73, and Ski8.

### Loss of Paf1 leads to PolII and CPSF100 accumulation

**Fig 6:**
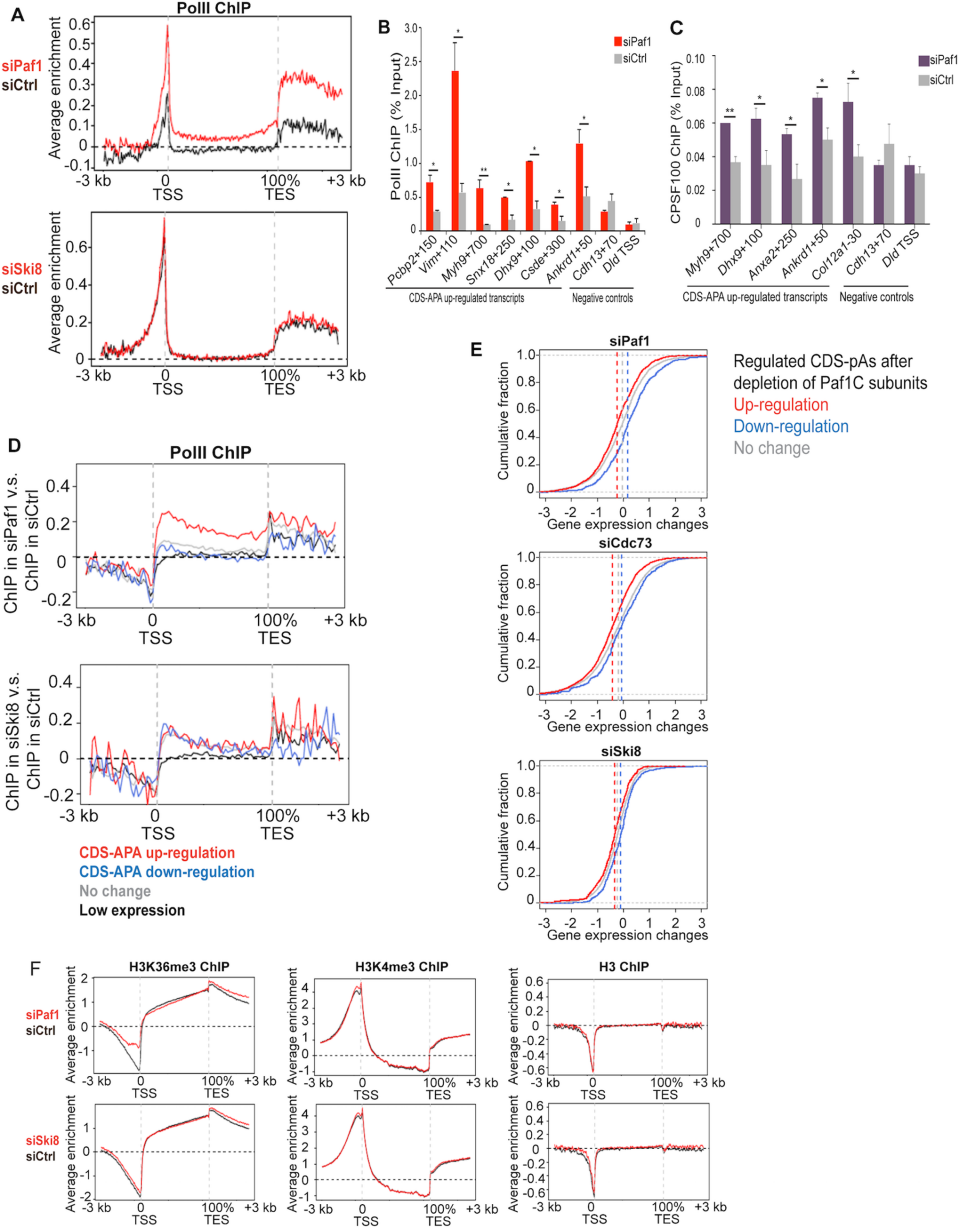
Paf1, but not Ski8, depletion leads to strong accumulation of PolII downstream of the TSS of genes with up-regulated intronic APA. **(A)** The average enrichment profiles of PolII after depletion of Paf1 or Ski8. A region from 3 kb upstream of TSS to 3 kb downstream of TES of all RefSeq genes was analyzed. (**B and C**) qChIP analysis and genome browser tracks demonstrate increased PolII and CPSF100 occupancy, respectively, immediately downstream of the TSS of genes that showed CDS-APA up-regulation after Paf1 depletion. In **(B)**, qChIP data obtained using two different anti-PolII antibodies were averaged. Genes with CDS-APA unaffected by Paf1 depletion are shown as controls. **(D)** The ratio of PolII ChIP-seq signals in Paf1 versus control siRNA-treated cells was plotted over genes that exhibited low or undetectable expression or that were unaffected or showed CDS-APA regulation after depletion of Paf1 or Ski8. A region from 3 kb upstream of TSS to 3 kb downstream of TES was analyzed. **(E)** Cumulative Distribution Function (CDF) curves indicate significant decrease in the levels of gene expression with up-regulated CDS-APA after subunit depletion. The *p*-values were calculated based on Wilcoxon Rank Sum Test by comparing the up- or down-regulated (red or blue line, respectively) to the unaffected CDS-APA events (gray line). Specifically, in Paf1 knock-down, *p* = 5x10^-20^ and *p* = 8x10^-23^, respectively; in Cdc73 knock-down, *p* = 4x10^-35^ and *p* = 1x10^-4^, respectively; and in Ski8 knock-down, *p* = 5x10^-7^ and *p* = 2x10^-8^, respectively. Median values were indicated as vertical dotted lines. The x-axis represents the log_2_ ratio of RPKM (reads per kilobase per million) values between knock-down and control samples. **(F)** The average enrichment profiles of H3K4me3, H3K36me3, and histone H3 density after depletion of Paf1 or Ski8 are shown. Regions from 3 kb upstream of TSS to 3 kb downstream of TES of all RefSeq genes were analyzed.

To further examine the distinct roles of Paf1 and Ski8 in transcription and 3’ end processing, we performed ChIP-seq on PolII, since its occupancy of gene bodies is a measure of transcriptional elongation. Interestingly, genome-wide comparison of its enrichment after Paf1 and control siRNA treatments indicated that PolII strongly accumulated on genic regions after Paf1 depletion (Fig 6A). By meta-gene analysis, we found that depleted cells exhibited substantial PolII enrichment over the entire gene body and up to several kb downstream of the TES (Fig 6A). In striking contrast, no differences in PolII enrichment were observed after Ski8 ablation (Fig 6A). We confirmed that PolII was indeed significantly enriched on a subset of genes using qChIP (Figs 6B and S6B). In addition, we found that the C/P factor CPSF100 was enriched on a subset of genes after Paf1 depletion (Fig 6C), although its protein levels were not altered (S6A Fig), confirming the enhancement of C/P activity within these TSS-proximal regions.

To further probe the correlation between changes in PolII occupancy and transcript shortening, we calculated the ratio of PolII ChIP-seq signals in Paf1 versus control siRNA-treated cells, focusing on groups of genes whose CDS-APA were up-regulated, down-regulated, or unaffected. Importantly, the highest ratio of the average PolII enrichment was observed on the group of genes that exhibited transcript shortening in the Paf1 knock-down, with the strongest accumulation occurring within the first ~10% of the gene body (Fig 6D). The prominent enrichment of PolII on genes exhibiting CDS-APA activation after Paf1 depletion prompted us to investigate the level of gene expression using RNA-seq data. Interestingly, cumulative distribution plots showed that genes that displayed CDS-APA activation were associated with decreased expression (all isoforms included), despite the elevated PolII occupancy (Fig 6E). These observations suggest that in the absence of Paf1, PolII progression is impeded, which could lead to increased pausing of PolII, a decrease in the rate of transcription, and stimulation of CDS-pA site usage. Given the association between PolII progression and specific histone modifications as well as the involvement of Paf1C in the deposition of histone modifications, we used ChIP-seq as an unbiased approach to determine whether histone modifications are globally altered after Paf1 ablation (Fig 6F). We did not examine H2Bub, given its wholesale loss upon silencing several Paf1C components (Fig 1C). However, we did not observe significant alterations in H3K4me3 or H3K36me3 genome-wide, consistent with results showing little impact on either modification globally (Fig 1C). Similarly, Ski8 knock-down produced no significant alterations in either of these marks. We conclude that Paf1C depletion is associated with the loss of H2Bub, but that other histone modifications, including H3K4me3 and H3K36me3, are not globally impacted, despite increased PolII occupancy.

## Discussion

**Fig 7:**
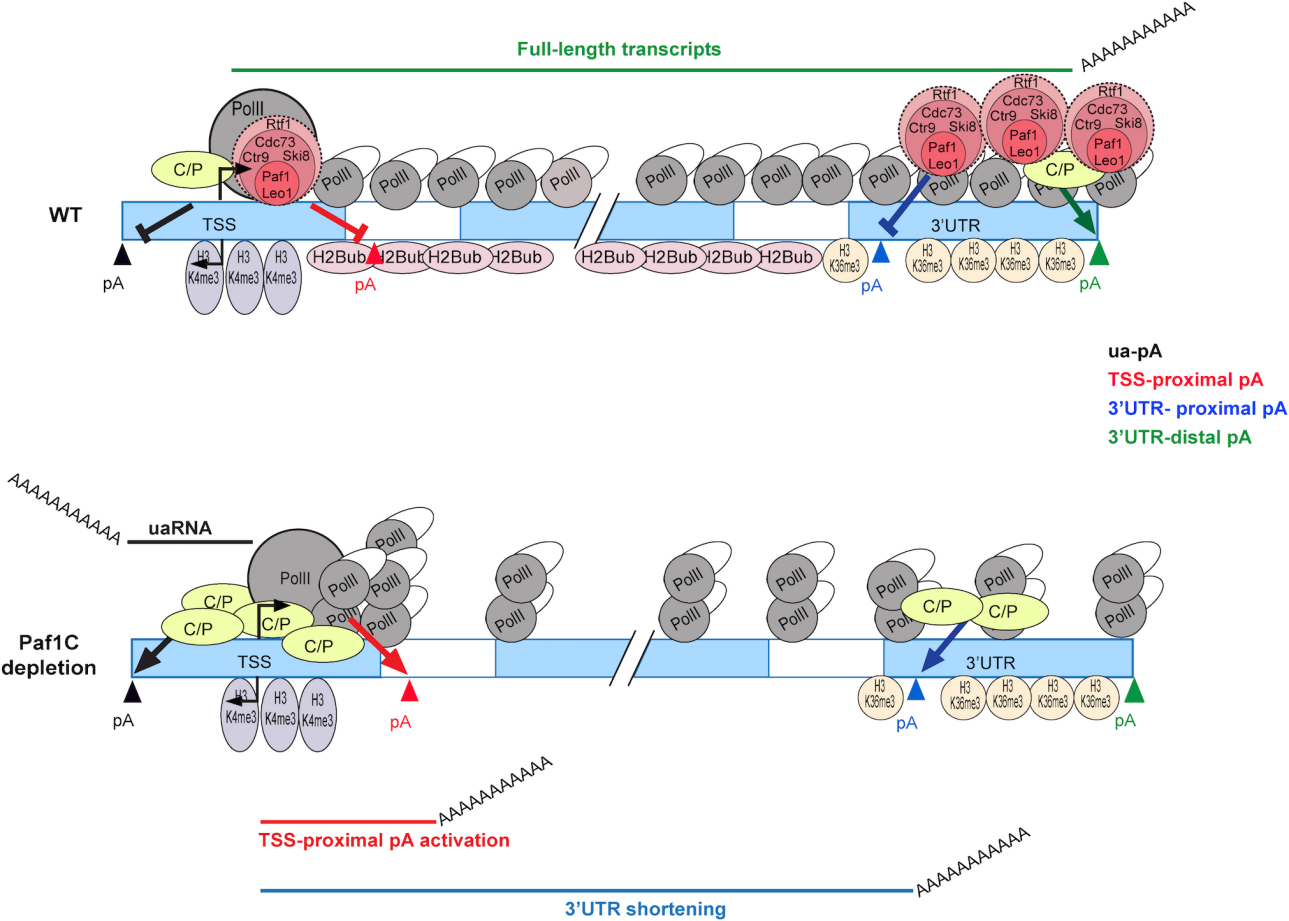
A model depicting the role of Paf1C in regulation of PolII progression and APA. Prevalent transcript shortening and increased production of upstream antisense transcripts occurs after depletion of Paf1C subunits. See text for details.

Here, we provide the first comprehensive analysis of mammalian PAF complex occupancy across the genome. In addition, our studies report the localization and function of this complex in normal mammalian cells, distinguishing it from others that were performed largely in yeast or human cancer cells. Based on our biochemical and genome-wide analyses in skeletal muscle cells, our data suggest that the PAF complex could be modular, with potentially distinct sub-complexes acting at discrete genic regions. Indeed, our data and those obtained in cancer cells suggest that Rtf1 could integrate into Paf1C transiently, forming a distinct module with alternative functions and the potential to regulate distinct genes (Figs 1 and 2; [7]). Moreover, our genome-wide analysis of histone modifications in Paf1- and Ski8-depleted cells led to the surprising conclusion that this factor may not play a predominant role in the deposition of H3K4 or H3K36 tri-methylation in mouse muscle cells. When taken together with our genome-wide ChIP-seq studies, these findings reinforce the notion that Paf1C could play distinct roles in yeasts and in mammalian cells, although additional studies will be needed to determine whether our findings can be generalized to other mammalian cell types.

Most importantly, our functional analyses suggest a novel model in which Paf1C plays a pivotal role in linking PolII progression with pA site selection (Fig 7) and suggest that accumulation of RNA polymerase II in the absence of Paf1C function could be mechanistically coupled to proximal pA usage. Links between PolII pausing and C/P have been reported (reviewed in [47]). For example, PolII pausing is associated with pA recognition and recruitment of C/P factors [48], and further, PolII pausing generates elevated levels of Ser2 phosphorylation of the CTD, which enables recruitment of C/P factors [49]. Our studies add a new dimension to an expanding list of functions performed by this complex transcription factor.

We observed increased cross-linking of PolII on gene bodies after depletion of Paf1, which would appear similar to observations described recently [50]. However, we observed decreased transcription of genes exhibiting CDS-APA activation after depletion of Paf1, suggesting that Paf1C functions as a positive elongation factor on these genes, reminiscent of findings in yeast, whereas Chen *et al*. [50] showed that ablation of Paf1 leads to increased transcription of many genes. Furthermore, Chen *et al*. observed the most pronounced accumulation of PolII at TSS-proximal regions after Paf1 depletion, whereas we observed enhanced PolII levels throughout the entire body of genes exhibiting changes in both classes of pA sites (3’UTR- and CDS-APA) (Figs 6D and S6C). There are numerous experimental differences that could explain these diverse observations. First, our conclusions are based on the group of genes exhibiting changes in APA, whereas Chen *et al*. focused on the set of genes enriched for paused PolII at the TSS. Second, Chen *et al*. performed their studies in human cancer cells, whereas our work involved mouse skeletal muscle. Apart from these technical differences, we believe that our findings could reflect distinct mechanisms, wherein Paf1C plays multiple roles in elongation: one function could include that described by Chen *et al*., namely, a negative role at the promoters of paused genes and a positive role over gene bodies. Thus, Paf1 could function through distinct mechanisms to mediate PolII pausing at the TSS and gene body. This could explain the distribution of PolII that we observe in Fig 6. It will be important to explore these mechanistic differences in the future by comparing distinct groups of genes (such as those that exhibit APA and pausing versus those that do not) using multiple cell types.

Since APA involves kinetic competition between different pA sites, it is conceivable that PolII elongation rate can impact pA choice. Indeed, alteration of PolII elongation rate has been implicated in APA [51]. A key question therefore is whether Paf1C-mediated APA regulation is a mere reflection of enhanced PolII pausing or slower elongation rate after Paf1 ablation. Evidence from previous studies and this work argue against this notion: First, we and others have observed robust interactions between C/P factors and Paf1C subunits in the absence of factor ablation [11, 38], suggesting an active role for Paf1C in pA choice. In addition, depletion of Ski8 led to changes in pA site usage within the 3’UTR without affecting the distribution of PolII (S6C Fig). These observations suggest that PolII pausing/elongation rate is not an obligatory feature of APA within our experimental system.

Intriguingly, CDK9, a kinase associated with P-TEFb and the Super Elongation Complex (SEC) known to phosphorylate Ser2 of the PolII CTD, is required for maintaining global levels of H2Bub, and its ablation led to altered 3’ end processing and enhanced read-through to an alternative pA site at replication-dependent histone genes, which are not normally polyadenylated [15]. The relationship between Paf1C and PolII elongation is complex, as noted previously, but it is clear that the activities of CDK9 and Paf1C are functionally intertwined. For example, suppression of CDK9 led to the loss of H2Bub and reduced Paf1 recruitment at a replication-dependent histone gene in mammalian cells [15]. Intriguingly, in fission yeast, mutations in PAF gene phenocopy *cdk9* loss-of-function, and PAF recruitment required CDK9 activity [16], in part because CDK9-mediated phosphorylation of Spt5, a component of the DRB-sensitivity inducing factor (DSF), promoted recruitment of Rtf1. Studies in HeLa cells further suggested that Paf1 plays an essential role in bridging CDK9 with PolII and recruiting CDK9 to genes [52], and ablation of the Ctr9 subunit of Paf1C led to reduced CDK9 recruitment [53]. In contrast, recent work in cancer cells and flies showed that depletion of Paf1 led to dramatic enhancement of CDK9 recruitment, coincident with release of paused PolII and increased phosphorylation of Ser2 on the CTD [6]. Since CDK9 phosphorylates PolII Ser2, and this modification stimulates recruitment of cleavage and polyadenylation factors, future studies will be needed to examine the interplay and potential mechanistic connections between H2Bub, CDK9 recruitment and activity, PolII progression, and Paf1C regulation of APA.

Our work has vastly extended previous studies to show that Paf1C is able to suppress both APA and upstream antisense transcription. We propose a model in which Paf1C regulates mRNA processing through direct control of RNA polymerase II progression during elongation and indirectly via expression of a cohort of cleavage and polyadenylation proteins (Fig 7). It will be interesting to examine the role of Paf1C in the regulation of upstream antisense transcription and to determine whether this activity is coordinated with histone modifications and exosome function.

Aspects of Paf1C-mediated APA regulation are reminiscent of recent results in which nuclear poly(A) binding protein (PABPN1), a factor involved in stimulating poly(A) polymerase and controlling poly(A) length, were ablated, triggering activation of TSS-proximal APA and shortening of 3’UTRs [43, 54, 55]. Interestingly, PABPN1 levels are increased during muscle regeneration, suggesting that this factor may have a role in repair of muscle tissue [56]. Moreover, trinucleotide repeat expansion of the PABPN1 gene is associated with oculopharyngeal muscular dystrophy (OMPD) [57]. OMPD mutations result in altered APA, suggesting that PABPN1 can suppress APA [54]. Future studies will be required to determine whether such a regulatory mechanism could be co-opted or subverted in physiological or patho-physiological settings, respectively.

## Materials and Methods

### Cell culture

C2C12 myoblasts (Sigma) were grown and induced to differentiate into myotubes as described [58]. Stable cell lines ectopically expressing Flag-tagged Cdc73, Rtf1, and Ski8 were generated by cloning cDNAs from Open Biosystems into pBabePuro as described [59]. For transient transfection of siRNA, cells were seeded at 10^4^ cells cm^−2^ for 48 h transfection. 5 μl RNAiMax in 2 ml medium and 50 nM siRNA were applied to cells by using fast-forward method. siRNA target sequences are listed below.

siPaf1: GCUAUGAGGAGAACUAUUU

siCdc73: GAUCCUACAUUGCGUACAA

siSki8: GGUGUUGAAUGUUGCGUUC

### Statistical analysis

All qChIP and RT-qPCR experiments were repeated at least twice independently except as noted, and the data are presented as mean ±SD and ± SEM for two and three or more repeats, respectively. Statistical significance was determined using Student’s t test, and is presented as *p < 0.05 or **p < 0.01.

### Antibodies used in this study

1. Ab: Paf1; Source: Affinity purified from Paf1 anti-serum [2]; Application: ChIP, IP and western blotting.
2. Ab: Leo1; Source: A300-175A-1, Bethyl; Application: ChIP, IP and western blotting.
3. Ab: Cdc73; Source: A300-170A (Lot # A300-170A-2) Bethyl; Application: ChIP, and IP.
4. Ab: Cdc73; Source: Kind gift from the Handa lab [4]; Application: western blotting.
5. Ab: Ctr9; Source: A300-BL679, Bethyl; Application: ChIP and western blotting.
6. Ab: Ctr9; Source: A301-395A, Bethyl; Application: IP and western blotting.
7. Ab: Rtf1; Source: A300-179A (Lot # A300-179A-1), Bethyl; Application: ChIP, IP and western blotting.
8. Ab: Ski8; Source: Affinity purified from Ski8 anti-serum [2]; Application: ChIP and western blotting.
9. Ab: RNA PolII; Source: Kind gift from the Bentley lab [60]; Application: ChIP.
10. Ab: RNA PolII; Source: 8WG16, Covance; Application: ChIP.
11. Ab: CPSF100; Source: Kind gift from the Manley lab; Application: ChIP and western blotting.
12. Ab: H3; Source: ab1791, Abcam; Application: ChIP and western blotting.
13. Ab: H3K4me3; Source: 39159, Active Motif; Application: ChIP and western blotting.
14. Ab: H3K36me3; Source: Ab9050, Abcam; Application: ChIP and western blotting.
15. Ab: H2Bub; Source: pw8810, Biomol International; Application: Western blotting.
16. Ab: α-tubulin; Source: T5168, Sigma; Application: Western blotting.

### Preparation of solubilized chromatin fraction and immunoprecipitation

A solubilized chromatin fraction was prepared as described [61]. Briefly, cell pellets were resuspended in buffer A (10 mM HEPES pH 7.9, 10 mM KCl, 1.5 mM MgCl2, 0.34 M sucrose, 10% glycerol, 1 mM dithiothreitol (DTT), and protease inhibitors (aprotinin, leupeptin, pepstatin A, and phenylmethyl sulphonyl fluoride)), supplemented with Triton X-100 (0.1%), and incubated on ice for 5 minutes. The nuclear pellet was separated from the cytoplasmic fraction, washed once with buffer A, and collected by centrifugation. Nuclei were lysed with buffer B (3 mM EDTA, 0.2 mM EGTA, 1 mM dithiothreitol (DTT), and protease inhibitors (aprotinin, leupeptin, pepstatin A, and phenylmethyl sulphonyl fluoride)). The solubilized chromatin fraction was separated from nucleoplasm by centrifugation at 2250 g for 4 minutes at 4°C, washed once with buffer B, and collected by centrifugation. For immunoprecipitations, the chromatin pellet was resuspended with binding buffer (20 mM Hepes pH7.9, 100 mM potassium chloride, 0.2 mM EDTA, 20% glycerol, 0.5 mM dithiothreitol (DTT), and 0.5mM AEBSF). 1 mg of protein was immunoprecipitated, and antibody-protein A sepharose beads were washed three times with buffer (50 mM Hepes pH7.9, 250 mM sodium chloride, 5 mM EDTA, 0.50% NP-40, and 10% glycerol) prior to SDS–PAGE and immuno-blotting.

### ChIP and ChIP-seq

ChIP was performed as described [62], followed by real-time quantitative PCR as described [63]. All primer sequences are listed below. ChIP-seq experiments in wild-type C2C12 and in Paf1C-depleted cells were performed as described [64] and [65], respectively. C2C12 histone modification (H3K4me3, H3K36me3, and H2Bub), PolII, and Spt6 data were previously published [63].

Primers for qChIP:

Spp1+1000F: TGAAATTGCCCTTTTCCTTG

Spp1+1000R: GCACCACTAGATCACCACCA

Ran+1500F: ATGCTCACGTGCTTCCTCTT

Ran+1500R: CTGGCCCATCAAAGTTCATC

Myog_+350_F: GAAAGTGAATGAGGCCTTCG

Myog_+350_R: AGGCGCTCAATGTACTGGAT

Myh3+25K F: GGTCCAGGAAGTGTCTCTGC

Myh3+25K R: CAGGAGGTCTTGCTCACTCC

mmGenedesert ChIP 5´: CCTCTGTAGCTGCCTCTCGT

mmGenedesert ChIP 3´: GTGTTGGGCAAGACTCTGCT

Pcbp2+150 F: CTCCCCTTTTCCCCTCAGTC

Pcbp2+150 R: AAGAAGGATGTCACGAGTGG

Vim+110F: TCCCTTGTTGCAGTTTTTCC

Vim+110R: GGTAGGAGGACGAGGACACA

Myh9+700F: TCCAAGGTTGAATGAGGTCAG

Myh9+700R: TCAGAGTGCGTGGGAAAAG

Snx18+250F: CTCGCTGCACAGGCTCAG

Snx18+250R: CGGGCGCTCTACGACTTTA

Dhx9+100F: CTTACCCTCCTCCGCTTTTC

Dhx9+100R: GTGTGTTCTTTCGCCGTTCT

Csde1+300F: AGGCGTCCCTTTTTCACC

Csde1+300R: CTGGCCTCACCTCCTCACTA

Ankrd1+50F: CATACCAGCTCCTCTACTCTCAG

Ankrd1+50R: CAGGGGTTCATCCACAAGAG

Cdh13+70F: GGCTCCCACGGAAAATATG

Cdh13+70R: GAGTTCTCGGCTGCATCTTG

Dld TSS F: GCTGAACGCCTGGTAAGACT

Dld TSS R: GACGAAGCGACCGGAAAG

Anxa2+250F: CAAAGTGTCCCGCAAGTGAC

Anxa2+250R: GAAACCTCAAGGGGAAGCAC

Col12a1-30 F: TACGAAACGCCTGAGAAGGT

Col12a1-30 R: TGCACACGTTCCCAAAAGTA

### Analysis of RNA and transcript length

Total RNA extraction and cDNA synthesis using anchored oligo d(T) primers have been described previously [64]. Primers designed to distinguish total transcripts and full-length transcripts are listed below. We calculated the relative abundance of full-length transcripts after depletion of Paf1C subunits as follows (adapted from [66]) First, the ratio between the full-length and total transcripts were determined individually for siPaf1C and siCtrl depleted cells by comparing qPCR threshold cycles, and then the ratio obtained from Paf1C-depleted cells was divided by the ratio obtained from siCtrl cells to determine the relative abundance of the full-length transcripts after depletion of Paf1C subunits.

For RNA-seq, ribosomal RNA was removed using RiboMinus Eukaryote System v2 (Ambion) (Invitrogen, A15026), and strand-specific cDNA library preparation and sequencing were performed as described [67]. 3’READS was performed as described [34].

Primers for RT-qPCR:

Tomm20_M-150F: TCAGGCAAACATGCAAATTC

Tomm20_M-150R: GGGTGTTCCATCTCAGCATT

Tomm20_L-150F1: TGGAAGAAGAAATGGGTGTG

Tomm20_L-150R: AATAATGCTGATGGCGCTCT

Ddx46_M-150F: TACCCCATACCCATCCAAAA

Ddx46_M-150R: CAAAATTCCAATGTCTTCCAGA

Ddx46_L-250F: CCCGGAAACTCCATACTGGT

Ddx46_L-250R: CACAAACAGAAAACGTGATACGA

Pdcd6ip_F-150F: AAATCAATAAACCAAAAGAGAAAGAAA

Pdcd6ip_F-150R: TAAGATGCAGTTGGGACAAAGA

Pdcd6ip_L-150F: TTTTCCCCCTAAGAGAAATGAA

Pdcd6ip_L-150R: TTATCACACCGGAGATTTTAGATT

B3galtl_F-150F: AGGGAAACTGGGAAAAATCG

B3galtl_F-150R: GGTGGGCTAAAATAGGCACA

B3galtl_L-150F: GGCTTTGTTCCTGCTGTCTT

B3galtl_L-150R: GGTGACTTGTTTAGAGGGCTTG

Gpc6_ex1_F: CTCTCACTGGCTCCCTCAGT

Gpc6_ex1_R: GTGTGTGCGTGGAGGTATGT

Gpc6_L_F2: CATGAGCAACACCTTAACGA

Gpc6_L_R1: TGCACACATTTATCCTGCAC

Arsk_F-130F: TGATGGACAGAAGCAGACACTT

Arsk_F-130R: CCGGATGAGCTAACAAACATT

Arsk_L-350F: GACTGGGTTGGGCTGCTA

Arsk_L-350R: ATTGGCAGAGAGATCCAAGG

Dld_F F: CATTGACTCGTTTCCAATATGA

Dld_F R: TTGACAGGAAGGAGCTGGAA

Dld_L F: TGGTGTCTTCATTCCCTGGT

Dld_L R: TTGCTGGCAGTTTAAGAGGT

Cdh13_ex1_F: GGCTCCCACGGAAAATATG

Cdh13_ex1_R: ACAGGGTGAGCGGAGTTCT

Cdh13_L_F: GTTTCAACCCCACACACAGT

Cdh13_L_R: GCGGAAGGCAAACTCAAA

Zfhx4_I F1: AAGTGTTAAAGGGTGCAAGGAA

Zfhx4_I R1: TTCTGTTTGGGTCATTCTTGTTT

Zfhx4_S F1: GATGTCTCAAATGCACCGTA

Zfhx4_S R1: CCAGAAGAATAAGTTCAAATACCATC

Col12a1_5'-Exon1F: CCGCTCCCTCTGTCACCT

Col12a1_5'-Exon1R: GGGAAGCCTGGTCTGCAT

Col12a1_L F1: TCAGTAGTTTTATTGAAAAATGCCTCT

Col12a1_L R1: AGAATTGCTGCTTTTATATTTTAACTG

Pcbp2 Exon5 F: GGTGCACGTATCAACATCTCA

Pcbp2 Exon5 R: AAGGCTTTGAAGATGGCATT

Pcbp2 Exon14 F: CGCCAAAATCAATGAGATCC

Pcbp2 Exon14 R: ATGGTAACCTGCCGATCAGT

Myh9_ex1_F: CTTCCGAGTGGACTTTCTCG

Myh9_ex1_R1: CTCAAGAACCTGACCTGCTG

Myh9_L_F: TGACGCTCAGTGGAAACATC

Myh9_L_R: AAAGGCGCTGTCATAAAGGA

Vim_ex1_F: CCTCATTCCCTTGTTGCAGT

Vim_ex1_R: GAGGACACAGACCTGGTAGACA

Vim_L_F: CAGCTTTCAAGTGCCTTTACTG

Vim_L_R: CGTCTTTTGGGGTGTCAGTT

Irs1_ex1_F: CGATTCCCGAGGCAAATTA

Irs1_ex1_R: GAGGAGAAGGAGGAGGGAGA

Irs1_L_F: CCAGACAGAATTGGGGGTAA

Irs1_L_R: TTTAGACGTGTTTCACTTTTCCAA

### RNA-seq data analysis

Reads were mapped to the mouse genome (mm9) using bowtie2 (local mode). Uniquely mapped reads with MAPQ score>10 were used. RefSeq gene model was used to calculate gene expression level. For protein-coding genes, only reads mapped to coding region were used to eliminate the effect of 3’UTR-APA in gene expression calculation. Reads per kilobase per million total uniquely mapped reads (RPKM) value was calculated to reflect gene expression level. Significantly regulated genes were selected based on these criteria: >1.4-fold difference of RPKM between Paf1C siRNA treated cells and siRNA control cells and *p-*value<0.01 (Fisher’s exact test).

### 3’READS and data analysis

3’READS library preparation was carried out as previously described [34, 43]. 3’READS reads were mapped to the mouse genome (mm9) using bowtie2 (local mode). Uniquely mapped reads (with MAPQ score > 10) that had at least two additional 5’ Ts not aligned to genome (presumably derived from the poly(A) tail) were named pA site supporting (PASS) reads and were used to calculate pA isoform expression. pA isoforms expressed below 5% of all isoforms of a gene were discarded. Significantly regulated APA events were identified by the Significance Analysis of Alternative Polyadenylation (SAAP) method [43]. 3’UTR-APA was based on comparing the two most abundant pA isoforms in the 3’UTR of a gene. CDS-APA was assessed by comparing all isoforms with pAs in upstream exon/intron regions versus all isoforms with pAs in the 3’-most exon of a gene. uaRNA regulation was based on comparing upstream antisense PASS reads within 2 kb from the TSS versus all sense strand reads. To study regulation of individual pA isoform, each isoform was compared to all others combined of the same gene. FDR = 0.05 (SAAP) was used to select significantly regulated APA events, unless noted otherwise. Samples were compared for the extent of APA regulation using the Global Analysis of Alternative Polyadenylation (GAAP) approach [43]. Briefly, one million and a half PASS reads were sampled from each sample to control the sequencing depth; SAAP analysis was carried out to identify significantly regulated APA events; and this process was repeated 20 times from which the mean and standard deviation of the number of significant events were calculated. For visualization of 3’READS data, only PASS reads were used to create bigwig format files and for visualization in the Integrative Genomics Browser (IGB).

### ChIP-seq data analysis

Reads were mapped to the mouse genome (mm9) using bowtie2. Reads non-uniquely mapped (MAPQ≤10) were discarded. Multiple reads mapped to same genomic loci (defined by chromosome, strand, start and end position) were collapsed to one.

To calculate enrichment score of a genomic locus in a meta-gene plot, the first nt at the 5’end of a read was counted for all genes in a group and converted to read per million total reads (RPM) value. The enrichment score was defined by log_2_ ratio of RPM between IP sample and input sample. The relative genomic location was binned (e.g., every 50 nt relative to TSS or every percent in gene body) before calculating enrichment score and then plotted in R.

The correlation of ChIP-seq data among different Paf1C factors was calculated based on the Pearson correlation of RPM values of four sub-regions of genes (i.e., TSS and TES upstream/downstream 1kb region). Only sub-regions with at least 50 reads in at least one sample were used for the calculation.

For visualization of ChIP-seq data, all uniquely mapped and collapsed reads were used. Reads were extended from its 5’end mapped position by 250 nts (reflecting the average fragment size in ChIP-seq DNA libraries) and converted to bigwig format files using an in-house perl script and bedGraphToBigWig program from UCSC genome browser. The bigwig files were visualized in IGB.

### Gene ontology (GO) analysis

GO annotations of genes were obtained from the NCBI Gene database, and GO analysis was carried out using the Fisher’s exact test [43].

### Accession numbers

ChIP-seq, RNA-seq and 3’READS data generated for this study were deposited under the primary accession GSE72574. Previously published data re-analyzed for this study include GSE44119 (Spt6 ChIP-seq, [68]), and GSE34960 (H2Bub ChIP-seq, [39]) and GSE25308 (PolII, and H3K4me3 ChIP-seq, [63]).

## Supporting Information

S1 Fig Rtf1 does not associate with Paf1C and confirmation of siRNA-mediated depletion of Paf1C subunits in mouse muscle cells. **(A)** Co-immunoprecipitation of Flag-tagged Cdc73 and Ski8, but not Rtf1, from nuclear extracts of myoblasts. **(B)** qRT-PCR verification of siRNA-mediated depletion of Leo1, Ctr9, and Rtf1 in myoblasts. Transcript levels of Paf1C subunits were normalized to *Gapdh*.
(TIF)

S2 Fig Correlation between the absence of Paf1C occupancy and low levels of gene expression, and distinct target regions of Paf1C subunits. **(A)** Metagene analysis shows average ChIP-seq enrichment profiles of indicated Paf1C subunits and PolII. A region from 3 kb upstream or downstream of the TSS or TES was analyzed. **(B)** *k*-means clustering (*k* = 10) of ChIP-seq data reveals common and distinct localization of Paf1C subunits, Spt6, histone modifications, and PolII, and correlation between Paf1C occupancy and levels of gene expression. Each row represents a single 4 kb genic region surrounding the TES. A heatmap representing RNA-seq analysis of myoblasts and myotubes. **(C)** qChIP validation of Cdc73 specific enrichment, and a representative genome browser track example of distinct target regions of Paf1C subunits. The y-axis represents normalized read density in reads per million (RPM). MB, myoblasts; MT, myotubes. **(D)** Cluster analysis of co-occupancy of biological replicates of Paf1C subunits on chromatin regions. Values are Pearson correlation coefficients. Correlations of RPM values encompassing genomic regions (TSS/TES upstream/downstream 1kb) were analyzed.
(TIF)

S3 Fig Depletion of Paf1C subunits leads to genome-wide de-regulation of gene expression. **(A)** GO analysis of Paf1C target genes. The y-axis indicates biological process. The x-axis represents significance score (-log_10_ *p*-value, Fisher’s exact test) [43]. MB, myoblasts; MT, myotubes. **(B)** Heatmap of RNA-seq data after depletion of Paf1, Cdc73, or Ski8. KD, Paf1C-depleted cells; Ctrl, control cells. **(C)** Genes exhibiting changes in expression after siRNA-mediated depletion of Paf1C subunits were classified according to GO analysis as described in S3A Fig. Genes that showed greater than 1.4-fold change in expression (*p*-value<0.01, Fisher's exact test) were analyzed. The y-axis indicates biological process, and the x-axis represents the significance score (-log_10_ *p*-value, Fisher’s exact test).
(TIF)

S4 Fig GO analysis of genes showing changes in APA after depletion of Paf1C subunits. Genes with significant APA regulation, SAAP method, FDR<0.05, were analyzed as in Fig 3B and 3C. GO analysis was performed as described in S3A Fig.
(TIF)

S5 Fig Chromatin occupancy of Paf1C is correlated with regulation of APA after depletion of Paf1C subunits. **(A)** Genome browser tracks depict enrichment of Paf1C subunits by ChIP-seq (top) for a control gene (*Dld*) that does not show changes in APA, as detected by 3’READS analysis, after depletion of Paf1C subunits. This gene serves as a control for comparison with genes (*Ddx46* and *Pcbp2*) that show changes in APA in Fig 4B. (**B**) The percentage of regulated intronic CDS pA sites in Paf1-, Cdc73- or Ski8-depleted cells. **(C)** The average enrichment profiles of Paf1, Cdc73, and Ski8 were plotted over genes that showed regulated or unaffected CDS-APA after depletion of Paf1, Cdc73 or Ski8. Genes exhibiting low levels of expression are also shown. Regions from 3 kb upstream of TSS to 3 kb downstream of TES were analyzed. **(D)** The average enrichment profiles of Paf1, Cdc73, and Ski8 were plotted over genes that showed shortening of 3’UTRs or that were unaffected after depletion of Paf1, Cdc73, or Ski8. Regions from 2 kb upstream or downstream of the two most regulated 3’UTR pA sites were analyzed.
(TIF)

S6 Fig PolII occupancy is unaffected by Ski8 depletion but is increased on genes exhibiting 3’UTR shortening after Paf1 depletion. **(A)** siRNA-mediated depletion of Paf1 did not affect the levels of CPSF100. siCtrl, non-specific control siRNA. **(B)** Representative genome browser tracks demonstrate increased PolII occupancy immediately downstream of the TSS of genes that showed CDS-APA up-regulation after Paf1 depletion. The gray rectangle shows a blown-up portion of the entire gene, *Myh9*, to the left. The y-axis represents normalized reads in reads per million mapped (RPM). Gene name and the maximum value of y-axis are indicated. siCtrl, non-specific control siRNA. **(C)** The ratio of PolII ChIP-seq signals in Paf1 or Ski8 versus control siRNA-treated cells was plotted over genes that were unaffected or showed 3’UTR shortening after depletion of Paf1 or Ski8. Regions from 2 kb upstream or downstream of the two most de-regulated 3’UTR pA sites were analyzed (a distance between the proximal and distal pAs greater than 1 kb was required).
(TIF)
