# Correction: PAF Complex Plays Novel Subunit-Specific Roles in Alternative Cleavage and Polyadenylation

**DOI:** 10.1371/journal.pgen.1005883

**Published:** 2016-02-18

**Authors:** Yan Yang, Wencheng Li, Mainul Hoque, Liming Hou, Steven Shen, Bin Tian, Brian D. Dynlacht

There is an error in the designations for equal authorship contributions. The first two authors, Yan Yang and Wencheng Li, should be noted as contributing equally to this work.
