# Supplementary figures and images for: PAF Complex Plays Novel Subunit-Specific Roles in Alternative Cleavage and Polyadenylation
Source: PLoS Genet. 2016 Jan 14;12(1):e1005794. doi: 10.1371/journal.pgen.1005794 (PMC4713055; doi:10.1371/journal.pgen.1005794)

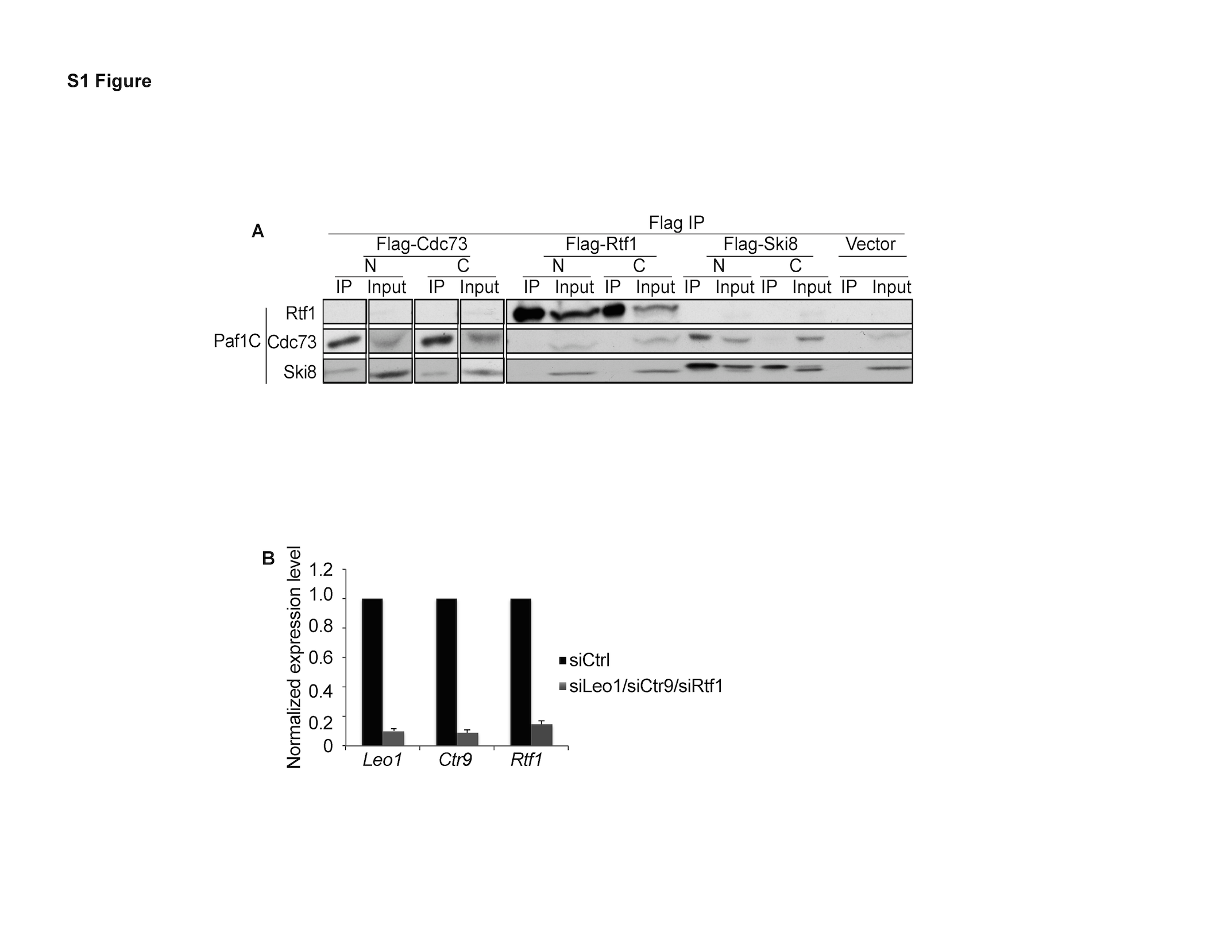

Supplement: S1 Fig — (A) Co-immunoprecipitation of Flag-tagged Cdc73 and Ski8, but not Rtf1, from nuclear extracts of myoblasts. (B) qRT-PCR verification of siRNA-mediated depletion of Leo1, Ctr9, and Rtf1 in myoblasts. Transcript levels of Paf1C subunits were normalized to Gapdh. (TIF) [file pgen.1005794.s001.tif]

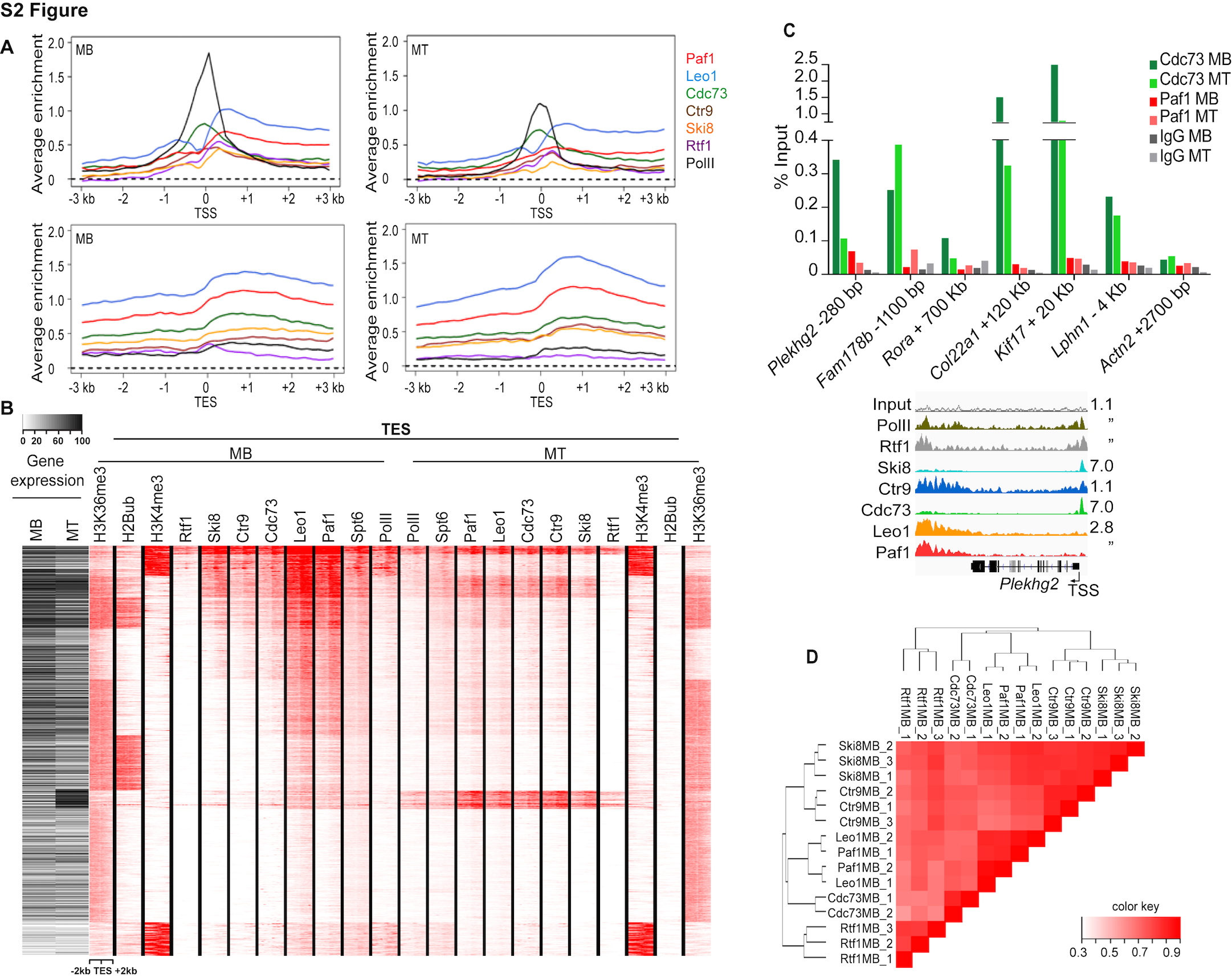

Supplement: S2 Fig — (A) Metagene analysis shows average ChIP-seq enrichment profiles of indicated Paf1C subunits and PolII. A region from 3 kb upstream or downstream of the TSS or TES was analyzed. (B) k-means clustering (k = 10) of ChIP-seq data reveals common and distinct localization of Paf1C subunits, Spt6, histone modifications, and PolII, and correlation between Paf1C occupancy and levels of gene expression. Each row represents a single 4 kb genic region surrounding the TES. A heatmap representing RNA-seq analysis of myoblasts and myotubes. (C) qChIP validation of Cdc73 specific enrichment, and a representative genome browser track example of distinct target regions of Paf1C subunits. The y-axis represents normalized read density in reads per million (RPM). MB, myoblasts; MT, myotubes. (D) Cluster analysis of co-occupancy of biological replicates of Paf1C subunits on chromatin regions. Values are Pearson correlation coefficients. Correlations of RPM values encompassing genomic regions (TSS/TES upstream/downstream 1kb) were analyzed. (TIF) [file pgen.1005794.s002.tif]

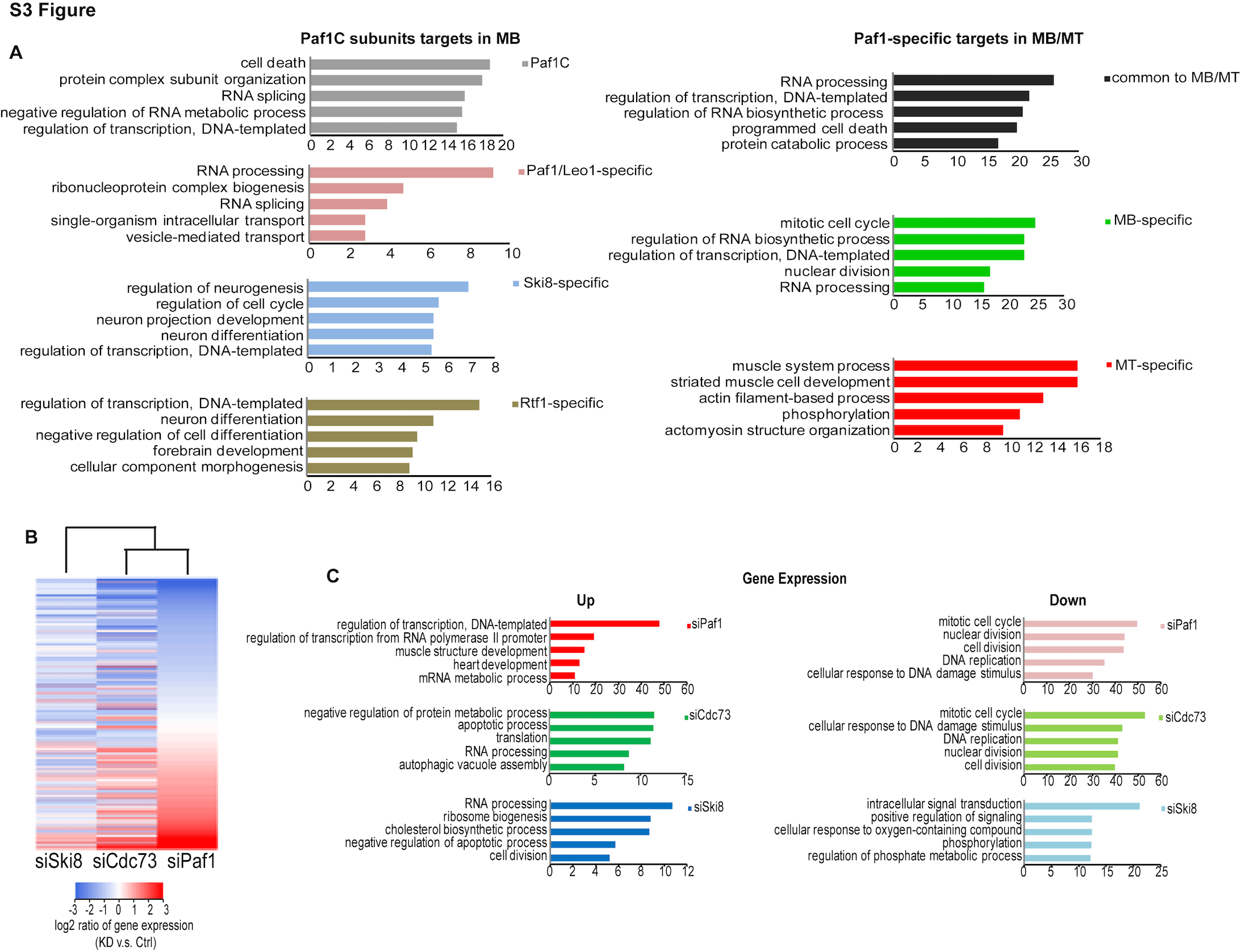

Supplement: S3 Fig — (A) GO analysis of Paf1C target genes. The y-axis indicates biological process. The x-axis represents significance score (-log10 p-value, Fisher’s exact test) [43]. MB, myoblasts; MT, myotubes. (B) Heatmap of RNA-seq data after depletion of Paf1, Cdc73, or Ski8. KD, Paf1C-depleted cells; Ctrl, control cells. (C) Genes exhibiting changes in expression after siRNA-mediated depletion of Paf1C subunits were classified according to GO analysis as described in S3A Fig. Genes that showed greater than 1.4-fold change in expression (p-value<0.01, Fisher's exact test) were analyzed. The y-axis indicates biological process, and the x-axis represents the significance score (-log10 p-value, Fisher’s exact test). (TIF) [file pgen.1005794.s003.tif]

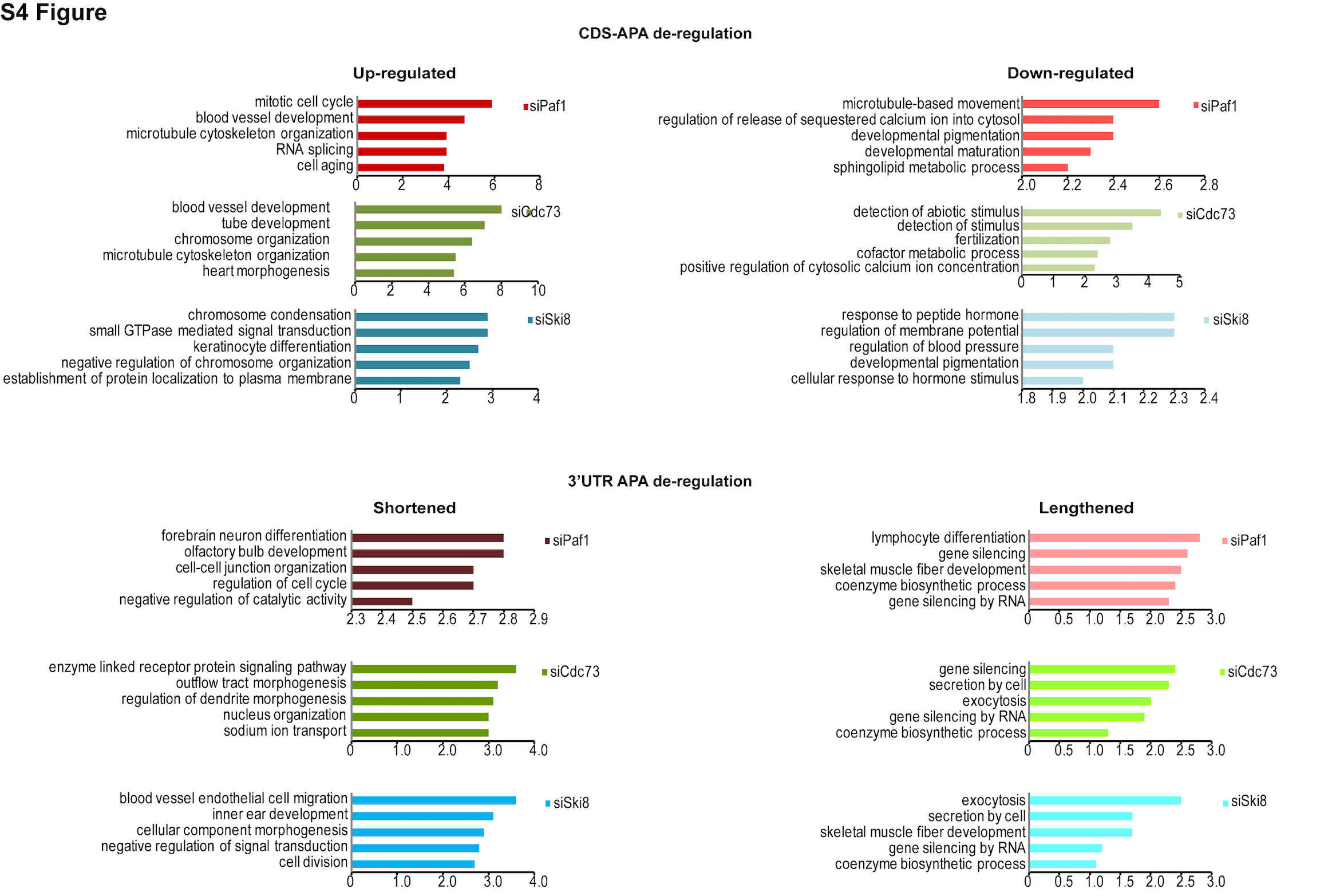

Supplement: S4 Fig — Genes with significant APA regulation, SAAP method, FDR<0.05, were analyzed as in Fig 3B and 3C. GO analysis was performed as described in S3A Fig. (TIF) [file pgen.1005794.s004.tif]

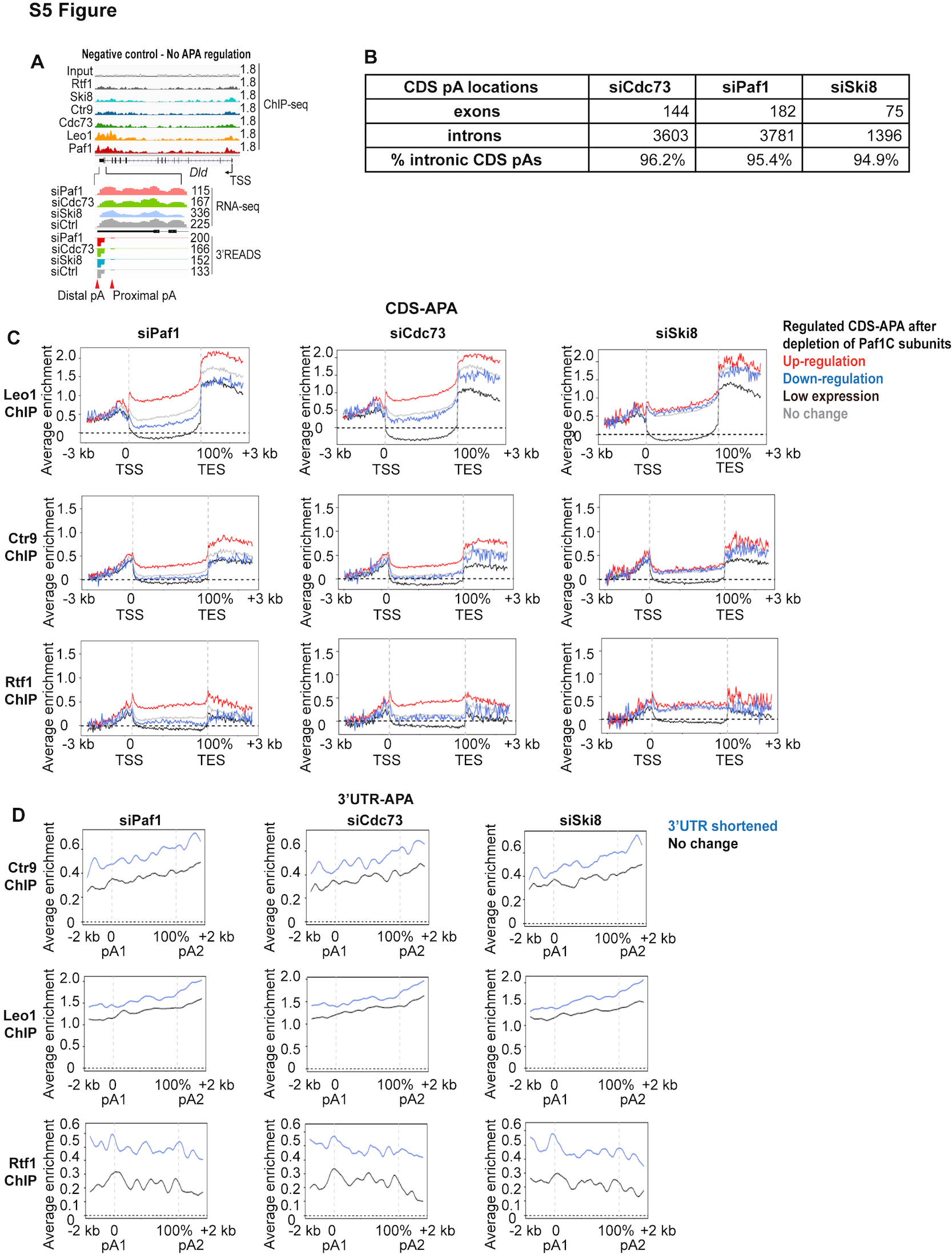

Supplement: S5 Fig — (A) Genome browser tracks depict enrichment of Paf1C subunits by ChIP-seq (top) for a control gene (Dld) that does not show changes in APA, as detected by 3’READS analysis, after depletion of Paf1C subunits. This gene serves as a control for comparison with genes (Ddx46 and Pcbp2) that show changes in APA in Fig 4B. (B) The percentage of regulated intronic CDS pA sites in Paf1-, Cdc73- or Ski8-depleted cells. (C) The average enrichment profiles of Paf1, Cdc73, and Ski8 were plotted over genes that showed regulated or unaffected CDS-APA after depletion of Paf1, Cdc73 or Ski8. Genes exhibiting low levels of expression are also shown. Regions from 3 kb upstream of TSS to 3 kb downstream of TES were analyzed. (D) The average enrichment profiles of Paf1, Cdc73, and Ski8 were plotted over genes that showed shortening of 3’UTRs or that were unaffected after depletion of Paf1, Cdc73, or Ski8. Regions from 2 kb upstream or downstream of the two most regulated 3’UTR pA sites were analyzed. (TIF) [file pgen.1005794.s005.tif]

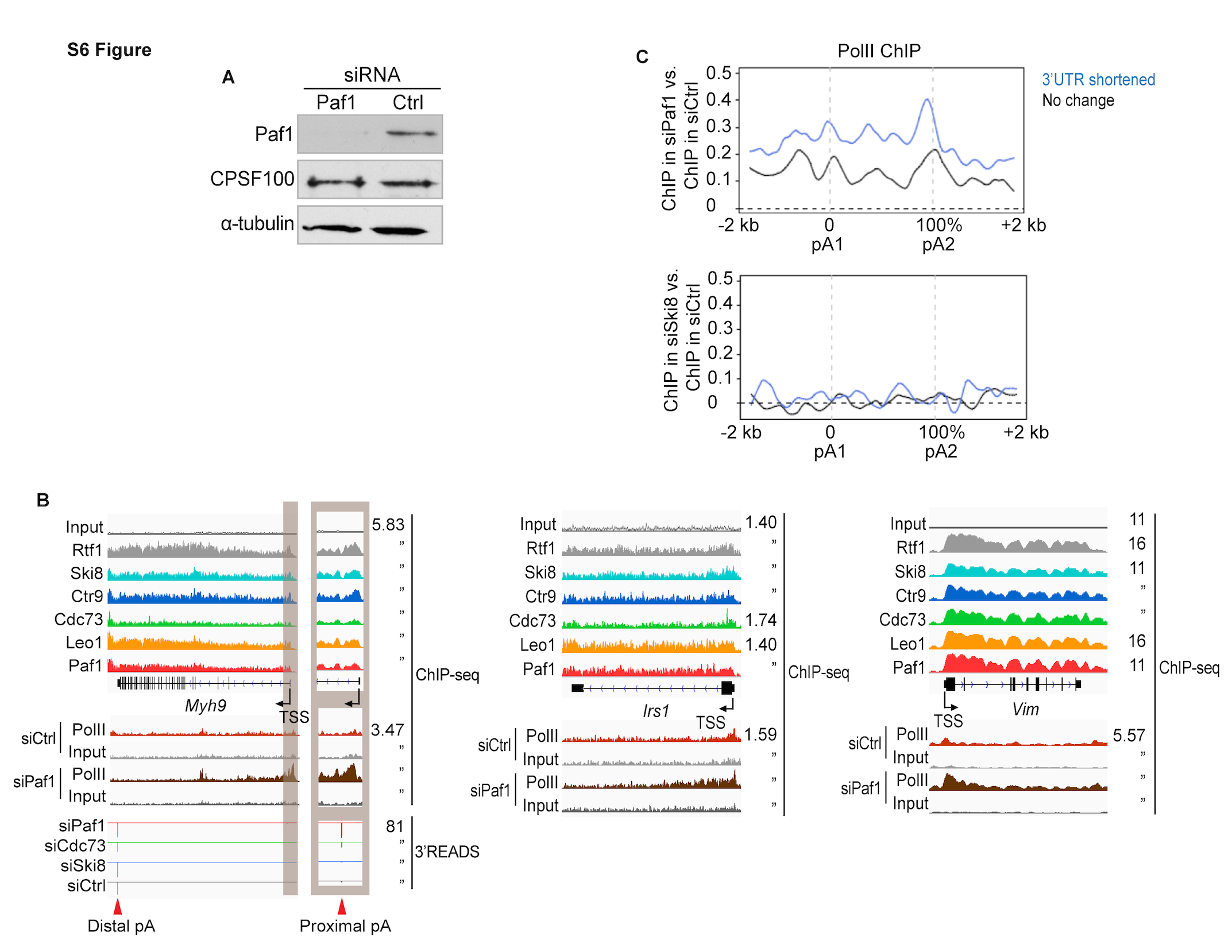

Supplement: S6 Fig — (A) siRNA-mediated depletion of Paf1 did not affect the levels of CPSF100. siCtrl, non-specific control siRNA. (B) Representative genome browser tracks demonstrate increased PolII occupancy immediately downstream of the TSS of genes that showed CDS-APA up-regulation after Paf1 depletion. The gray rectangle shows a blown-up portion of the entire gene, Myh9, to the left. The y-axis represents normalized reads in reads per million mapped (RPM). Gene name and the maximum value of y-axis are indicated. siCtrl, non-specific control siRNA. (C) The ratio of PolII ChIP-seq signals in Paf1 or Ski8 versus control siRNA-treated cells was plotted over genes that were unaffected or showed 3’UTR shortening after depletion of Paf1 or Ski8. Regions from 2 kb upstream or downstream of the two most de-regulated 3’UTR pA sites were analyzed (a distance between the proximal and distal pAs greater than 1 kb was required). (TIF) [file pgen.1005794.s006.tif]
